# The Troublesome Ticks Research Protocol: Developing a Comprehensive, Multidiscipline Research Plan for Investigating Human Tick-Associated Disease in Australia

**DOI:** 10.3390/pathogens11111290

**Published:** 2022-11-03

**Authors:** Amanda D. Barbosa, Michelle Long, Wenna Lee, Jill M. Austen, Mike Cunneen, Andrew Ratchford, Brian Burns, Prasad Kumarasinghe, Rym Ben-Othman, Tobias R. Kollmann, Cameron R. Stewart, Miles Beaman, Rhys Parry, Roy Hall, Ala Tabor, Justine O’Donovan, Helen M. Faddy, Marjorie Collins, Allen C. Cheng, John Stenos, Stephen Graves, Charlotte L. Oskam, Una M. Ryan, Peter J. Irwin

**Affiliations:** 1 Centre for Biosecurity and One Health, Harry Butler Institute, Murdoch University, Murdoch, WA 6150, Australia; 2 CAPES Foundation, Ministry of Education of Brazil, Brasilia 70040-020, DF, Brazil; 3 Australian Rickettsial Reference Laboratory, University Hospital Geelong, Geelong, VIC 3220, Australia; 4 The App Workshop Pty Ltd., Perth, WA 6000, Australia; 5 Emergency Department, Northern Beaches Hospital, Sydney, NSW 2086, Australia; 6 School of Medicine, Macquarie University, Sydney, NSW 2109, Australia; 7 Sydney Medical School, Sydney University, Camperdown, NSW 2006, Australia; 8 School of Medicine, University of Western Australia, Crawley, WA 6009, Australia; 9 College of Science, Health, Education and Engineering, Murdoch University, Murdoch, WA 6150, Australia; 10 Western Dermatology, Hollywood Medical Centre, Nedlands, WA 6009, Australia; 11 Telethon Kids Institute, Nedlands, WA 6009, Australia; 12 CSIRO Health & Biosecurity, Australian Centre for Disease Preparedness, Geelong, VIC 3220, Australia; 13 PathWest Laboratory Medicine, Murdoch, WA 6150, Australia; 14 Pathology and Laboratory Medicine, Medical School, University of Western Australia, Crawley, WA 6009, Australia; 15 School of Medicine, University of Notre Dame Australia, Fremantle, WA 6160, Australia; 16 School of Chemistry and Molecular Biosciences, University of Queensland, St. Lucia, QLD 4072, Australia; 17 Australian Infectious Diseases Research Centre, Global Virus Network Centre of Excellence, Brisbane, QLD 4072, Australia; 18 Queensland Alliance for Agriculture and Food Innovation, Centre of Animal Science, University of Queensland, St. Lucia, QLD 4072, Australia; 19 Clinical Services and Research, Australian Red Cross Lifeblood, Sydney, NSW 2015, Australia; 20 School of Health and Behavioural Sciences, University of the Sunshine Coast, Petrie, QLD 4502, Australia; 21 School of Psychology, Murdoch University, Murdoch, WA 6150, Australia; 22 School of Public Health and Preventive Medicine, Monash University, Clayton, VIC 3800, Australia; 23 Infection Prevention and Healthcare Epidemiology Unit, Alfred Health, Melbourne, VIC 3004, Australia; 24 Health Futures Institute, Murdoch University, Murdoch, WA 6150, Australia

**Keywords:** DSCATT, ticks, tick-borne infection, tick-borne disease, Lyme disease-like illness, biomarkers

## Abstract

In Australia, there is a paucity of data about the extent and impact of zoonotic tick-related illnesses. Even less is understood about a multifaceted illness referred to as Debilitating Symptom Complexes Attributed to Ticks (DSCATT). Here, we describe a research plan for investigating the aetiology, pathophysiology, and clinical outcomes of human tick-associated disease in Australia. Our approach focuses on the transmission of potential pathogens and the immunological responses of the patient after a tick bite. The protocol is strengthened by prospective data collection, the recruitment of two external matched control groups, and sophisticated integrative data analysis which, collectively, will allow the robust demonstration of associations between a tick bite and the development of clinical and pathological abnormalities. Various laboratory analyses are performed including metagenomics to investigate the potential transmission of bacteria, protozoa and/or viruses during tick bite. In addition, multi-omics technology is applied to investigate links between host immune responses and potential infectious and non-infectious disease causations. Psychometric profiling is also used to investigate whether psychological attributes influence symptom development. This research will fill important knowledge gaps about tick-borne diseases. Ultimately, we hope the results will promote improved diagnostic outcomes, and inform the safe management and treatment of patients bitten by ticks in Australia.

## 1. Introduction

Tick-borne diseases (TBDs) are an increasing burden for human and animal health globally [1,2]. While extensive research has been conducted into the epidemiology, pathophysiology and clinical outcomes of medically important TBDs in North America, Europe and parts of Asia, there is still relatively limited knowledge about the extent and impact of tick-related medical problems in Central America, Oceania, vast areas of Asia and most of Africa and South America [3,4,5,6,7,8,9].

In Australia, infectious TBDs known to be locally acquired in Australia comprise rickettsioses caused by *Rickettsia* spp. (Queensland Tick Typhus, Flinders Island Spotted Fever and Australian Spotted Fever) and *Coxiella burnetii* (Q fever), although the latter is not considered primarily tick-borne [5]. Three species of native ticks are known to transmit these bacterial infections to people: the paralysis tick (*Ixodes holocyclus*) which is associated with Queensland Tick Typhus and Q fever, the ornate kangaroo tick (*Amblyomma triguttatum*) which transmits *Coxiella burnetii*, and the southern reptile tick (*Bothriocroton hydrosauri*) which has been implicated in the transmission of Flinders Island Spotted Fever [5]. Non-infectious TBDs associated with Australian ticks include allergies, e.g., tick anaphylaxis, mammalian meat allergy (MMA), paralysis, and autoimmune disease [10,11].

The diseases listed above have well-defined pathologies, approved diagnostic pathways and are generally relatively acute in their progression. Despite this however, many Australians have reported suffering from an ill-defined illness following a tick bite [4,12,13,14]. It is unclear whether this illness results from infection by one or more of the organisms mentioned above, is due to hitherto unrecognised infectious pathogens, or arises from other non-infectious perturbations of the immune or neurological systems. In 2018, following parliamentary inquiries [15], the Australian Government Department of Health proposed the term “Debilitating Symptom Complexes Attributed to Ticks” (DSCATT) to acknowledge this patient group and the multifaceted illness of hitherto unknown aetiology [14]. The true scale of this problem is difficult to estimate, in part because: (1) an appropriate case definition does not exist, (2) with the exception of Q fever and tularemia (not currently known to be tick-transmitted in Australia), tick-borne infections are not notifiable, and (3) data on tick bites and their sequelae are not systematically collected or reported [5]. It is still unknown whether these symptoms develop only in a subset of patients bitten by ticks, their incidence, and time course.

A clinical pathway commissioned by the Australian Department of Health was developed to support decision-making on differential diagnosis and referral avenues for patients presenting with either new onset or unresolved debilitating symptoms, with or without a history of tick bites and that cannot be attributed to another condition [16]. According to this document, the most common symptoms of DSCATT patients are fatigue, disordered thinking, sensory disturbance, arthralgia, and headache. Self-reported symptoms also included myalgia, rash, mood and visual disturbances, dizziness, pain, fever, nausea, palpitations, insomnia, seizures, diarrhoea, tremor, and personality change [16].

More recently, a case series comprising a cohort of 29 patients experiencing DSCATT reported a clinical syndrome involving fatigue, headache, and arthralgia, at times resulting in severe physical impairment and financial stress. Anxiety, depression, and psychosocial stressors were also common. The authors did not find convincing evidence of infective causes among the patients studied [17].

Whilst an infectious aetiology of DSCATT is suspected, this hypothesis has not yet been tested robustly. With the exception of rickettsioses and Q fever, conclusive evidence of other locally acquired infectious aetiological agent(s) of tick-borne illness after a tick bite in Australia remains elusive. For example, autochthonous human cases of borrelioses, anaplasmosis, ehrlichioses, and tick-borne encephalitis, which are highly prevalent throughout the northern hemisphere, have not been diagnosed in Australia. Lyme disease (LD) caused by spirochetes belonging to the bacterial complex *Borrelia burgdorferi* sensu lato (s.l.) has for decades been the focus of debates between patient advocacy groups and their healthcare providers [15].

Current research suggests that LD is not locally acquired, since the aetiological agent(s) of LD (and other northern hemisphere tick-borne pathogens) have not been discovered within tick-wildlife ecologies on the Australian continent. Supporting evidence includes: (1) Vectors of LD overseas (e.g., *Ixodes scapularis*, *I. pacificus*, and *I. ricinus*) that carry *Borrelia burgdorferi* s.l. and other northern hemisphere tick-borne pathogens are not present in Australia [13,18,19,20,21,22,23]; (2) Studies employing culture to isolate *B. burgdorferi* s.l. from patients with presumed autochthonous LD in Australia were unsuccessful [13]; (3) People with no travel history with suspected LD have tested negative for *B. burgdorferi* s.l. by Australian accredited pathology laboratories. In contrast, such laboratories regularly diagnose LD in returnees from endemic areas who fulfill the clinical case definition for LD with *B. burgdorferi* s.l. infections [12,13]; and (4) A study involving dogs as sentinels living in tick ‘hot spots’ did not detect serological evidence of exposure to *B. burgdorferi* s.l. antigens [24]. 

Why has it been so difficult to diagnose autochthonous instances of the TBDs within Australia that are readily detected in other parts of the world? The answer may be that the native tick fauna are unique due to the continent’s long geological isolation since the Gondwanan break-up some 130–135 million years ago [25,26]. Indeed, recent metagenomic and metatranscriptomic analyses have revealed a diverse microbiota in Australian ticks comprising known bacterial, viral, and protozoal genera and novel species that are related to, yet phylogenetically distinct from, northern hemisphere tick-borne pathogens [19,27,28,29,30,31,32,33,34,35]. In other words, separate evolutionary and ecological pathways have resulted in not only a unique mammalian fauna but may also have impacted tick-associated microbial communities on the Australian continent.

Within this framework, an investigation of the disease causation (if any) by these organisms is urgently needed and constitutes the fundamental priority of our current research into tick-associated illness in Australia. New technologies hold promise for the study of complex tick-associated pathologies and to better understand the trajectory of highly variable symptomology [36]. These technologies can be called upon to investigate TBDs while considering the complex interactions between a triad of players, namely: the host immune response, the biting tick, and inoculated microbes. However, rigorous studies of the long-term effects of tick bite poses significant challenges, even when pathogens are recognised. We designed a longitudinal study with three control groups to investigate associations between microorganisms transmitted to patients during a tick bite and the development of clinical, pathological, immunological, and psychological abnormalities. We believe this is the first study conducted worldwide to report nationwide over a one-year period on the somatic and psychological effects of tick bite. We describe here in detail the study design and cross-disciplinary applications of multi-omics, metagenomics technologies, and psychometric analyses to better understand symptoms after a tick bite.

## 2. Aims

The major aim of this study is to provide an evidence-based understanding of the cause(s) of human tick-associated illness in Australia and to elucidate the cause(s) of DSCATT. The research sub-aims are to:

1. Identify and characterise microbes inoculated into the skin of humans during tick attachment;
2. Characterise the clinical features of tick bite, with and without transmission of microorganisms;
3. Describe host haematological, serum chemistry, and immunological responses to tick bite;
4. Ascertain if there is a relationship between baseline psychological profiles and the development of symptoms after tick bite; and
5. Develop molecular and serological diagnostic tests appropriate to Australian conditions.

## 3. Hypotheses

1. Following a tick bite, some patients will develop acute (within a month post-bite) or later onset (>1 month) symptoms (dermatological, rheumatological, neurological, and cardiac abnormalities); psychological changes (assessed by psychometric testing) or symptoms fulfilling the case definitions of fibromyalgia [37], chronic fatigue syndrome (CFS) [38], or myalgic encephalomyelitis (ME) [39]; and perturbations in routine pathology, serology, and immune profiles.
2. Microbial species found consistently in ticks, paired skin biopsies, and/or blood samples of patients developing illness after tick bite, and not in the controls, likely represent candidates for the aetiologic agent(s) of tick-associated illness and, potentially, DSCATT in Australia.

## 4. Methods

### 4.1. Study Design Overview

**Figure 1:**
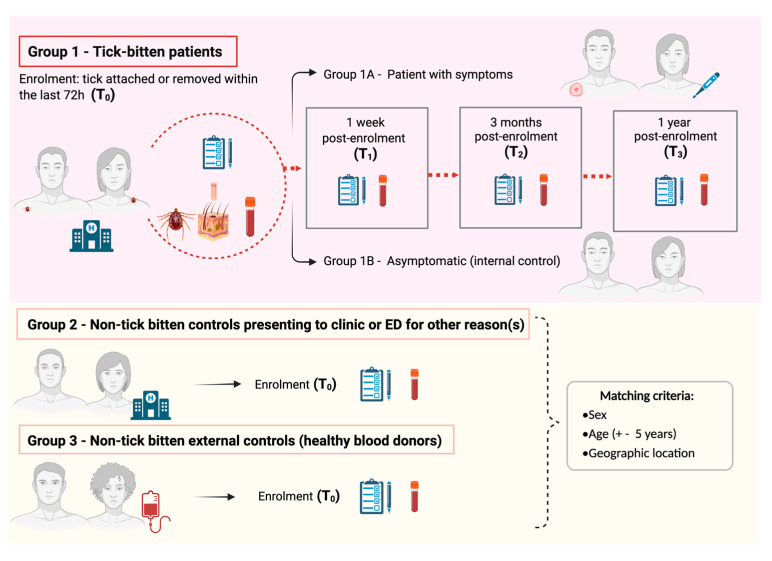
Overview of the Troublesome Ticks-DSCATT study scientific design. Image not drawn to scale.

This is an Australian nationwide, four-year longitudinal cohort study of human patients with a tick bite, which includes one internal and two matched external control groups. An overview of the study’s scientific design is represented in Figure 1. This is the most appropriate design to test our hypotheses as it allows causal inference, as well as characterisation of the pathophysiology of acute and chronic clinical presentations post-tick bite. This scientific design has been widely used in infectious disease research; for example, it has recently been adopted to determine pathophysiological mechanisms implicated in chronic sequelae of COVID-19 and Lyme borreliosis overseas [40,41].

People with a tick attached to their skin or who have removed a tick within the previous 72 h are invited to enrol in the study. Exclusion criteria comprise children (<18 years old), pregnant women; patients with coagulopathy or receiving anticoagulant therapy (except aspirin); and/or previously diagnosed with one or more of the following: ME, CFS, fibromyalgia, LD, or chronic “Lyme disease-like” illness (sometimes referred to as DSCATT).

In summary, at the time of enrolment (T_0_), consenting tick-bitten patients (Gp1) at a participating Emergency Department (ED) or general practice are asked to provide demographic information and to answer questions about their health and wellbeing, followed by the collection of the tick, a skin biopsy at the tick bite site, and blood samples. Skin biopsies collected from the site of tick attachment are considered valuable clinical samples for the diagnosis of TBDs [42,43] and vector-borne protozoan infections in other parts of the world (e.g., [44]). Molecular analyses of skin biopsies in addition to blood samples are crucial to increase the chances of pathogen detection, given low pathogen burden coupled with transient bacteraemia in blood samples [45].

Enrolment in regional areas is arranged through the patient’s general practitioner (GP). A sub-cohort of patients provide a consented control biopsy collected from the contralateral (healthy) skin site for spatial phenotyping studies (details provided later in: “Spatial phenotyping”). Patients are recruited nationwide with a focus on areas of known tick activity in proximity to humans, e.g., coastal New South Wales (NSW), coastal Queensland (Qld), and south-western Western Australia (WA). Informed by preliminary studies showing >1500 patients presenting with ticks attached presented to Northern Sydney hospitals alone over 20 months in 2016 and 2017 (unpublished data), our estimated patient enrolment was approximately 300 patients per year, nationwide, over three annual tick seasons (August to March 2020/21, 2021/22, and 2022/23).

Enrolled tick-bitten patients attend follow-up blood collection appointments at a local pathology collection centre, and complete follow-up questionnaires, at one week (T_1_), 3 months (T_2_), and 12 months (T_3_) post-enrolment. Based on responses and routine pathology results, Gp1 is sub-divided into a nested case–control group to include participants who have symptoms (local and/or systemic) following a tick bite (cases; Gp1A) and those that do not develop symptoms (internal controls; Gp1B). Patients in Gp1B who subsequently develop illness and symptoms (local or systemic) within 1 month of a tick bite are reassigned from Gp1B to Gp1A. 

A working case definition developed by our team will be applied as follows: (1) *Acute onset* (symptoms develop within 3 months of tick bite) of any one or more of (a) cutaneous reactions (inflammation at tick bite-site, eschar, erythema chronicum migrans, lymphocytoma); (b) rheumatological (asymmetric large joint oligoarthritis); (c) neurological (nerve palsies, meningism, impairment of consciousness); (c) cardiac abnormalities (heart-block, myocarditis); (d) systemic signs (fever, influenza-like symptoms); and/or (d) psychological changes (assessed by psychometric testing). (2) *Chronic onset* (later onset > 3 months) with clinical abnormalities described above or symptoms fulfilling the case definition of ME, fibromyalgia, or CFS. Based on limited tick-bite illness data overseas [46] and clinical observations following *I. holocyclus* bites at the Sydney hospitals, it is estimated that approximately 10% of patients with a tick bite will develop local or systemic symptoms consistent with this case definition for acute or chronic illness.

Two groups of non-tick-bitten external controls (matched by sex, age ±5 years, and geographical location) are also recruited into the study. Gp2 consists of patients presenting to ED or GP clinics for a reason other than a tick-bite (e.g., trauma, cardiac or respiratory illness). These situational controls are recruited ideally within 24 h of the enrolment of a Gp1 patient, and ensure that immunological signatures identified in Gp 1 are specific to TBDs and not just a marker of acute illness. Moreover, they serve to control for psychometric profiles linked with being unwell and/or visiting an ED or GP.

Gp3 is the primary control group for comparison with patients exposed to ticks. It comprises healthy blood donors recruited through Australian Red Cross Lifeblood (Lifeblood) from a close geographic location to Gp1 patients (determined by postcode of residence). Selected blood donors are also matched to Gp1 patients by sex and age (±5 years). Similar demographic, health and wellbeing questionnaires are filled by Gp2 and Gp3 participants, and blood samples (only) are collected from these 2 study control groups. The same exclusion criteria outlined for Gp1 also apply to Gp2 and Gp3 study participants.

External controls (Gps 2 and 3) are particularly useful if, for example, samples are positive for microbes in both cohorts of the tick-bitten group and would provide evidence of subclinical exposure to tick organisms.

### 4.2. Study Advertising Campaign

Materials are designed to promote and disseminate information about the present study to the general public, especially at the start of ‘tick seasons’ (August–February in Australia). These include posters (Figure S1), flyers (Figures S2 and S3) and customised recruitment animated videos created in Vyond [47] (Videos S1 and S2). To further encourage engagement from the community and increase enrolments, the study is advertised via social media (Facebook, Twitter, Instagram), radio interviews (e.g., ABC Health Report [48], ABC Radio Perth [49]), newspapers, e-mail, visits to national parks, clinics (medical and veterinary), libraries, pharmacies, community centres, and relevant associations whose members are deemed at risk of tick exposure (e.g., Australian Association of Bush Regenerators).

A study website [50] is designed to provide key information for participants and doctors, containing frequently asked questions, additional resources on tick bites in Australia, and a ‘contact us’ page. Additionally, a study hotline is made available for the duration of the study to facilitate immediate communication from the public with the project manager.

### 4.3. Participant Enrolment Process Overview

The standard study enrolment protocol comprises the following steps:

1. Identification of an eligible participant (tick killed in situ if still attached);
2. Provision of information about the study, consenting, completion of questionnaires;
3. Biospecimen collection and couriering; and
4. Arrangements for follow-up visits (Gp1 only).

An animated video describing this protocol was created using Vyond (see Video S3) and distributed to all participating medical staff to ensure consistency between enrolment sites, compliance with ethical requirements, and biospecimen and data integrity. 

### 4.4. Participant Enrolment Process: The Troublesome Ticks Study Portal

When a tick-bitten patient presents at an ED, or GP clinic, during triage, the tick (if still attached) is killed (i.e., frozen) in situ using an ether-containing spray, e.g., Medi Freeze or Tick Off Spray^®^ (PharmaCare Laboratories Pty Ltd., Warriewood, NSW, AU) as recommended by Australasian Society of Clinical Immunology and Allergy [51]. Potential participants are provided with information about the study and asked to consider enrolment prior to tick removal (details provided in the following section: “Biospecimen collection, transportation and processing”). Control individuals are similarly informed about the study and what their participation involves, prior to enrolment.

**Figure 2:**
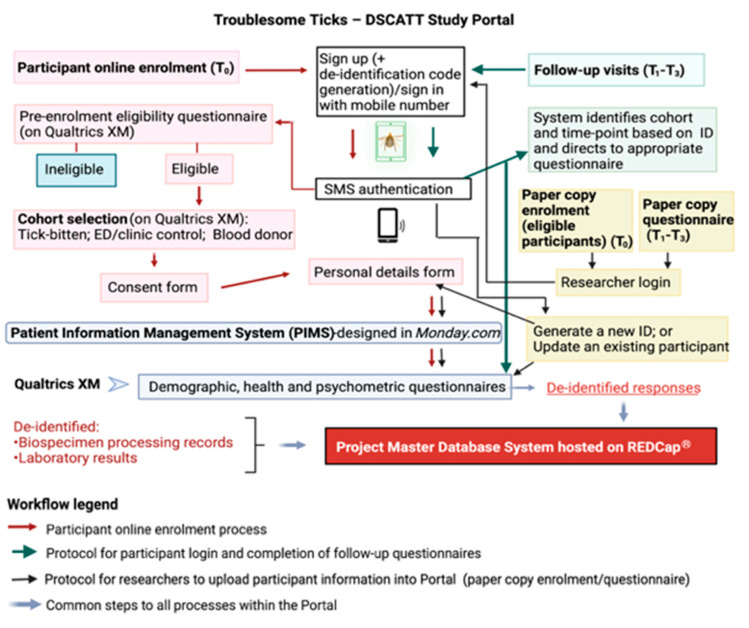
The Troublesome Ticks—DSCATT Study Portal and Database Systems.

The consent process is conducted either electronically (using an iPad provided) or by hard copy (paper) documents. If enrolling electronically, individuals are provided access to a study portal (the portal) designed and created specifically for this research to ensure efficient and secure data capture and management (Figure 2). A screenshot of the portal landing page design can be seen in Figure S4. Using Amazon Web Services (AWS), the portal integrates three operating systems: a task management platform (Monday.com, Tel Aviv, Israel), a survey platform (Qualtrics XM, Seattle, WA, USA), and a data storage and management platform (REDCap, Research Electronic Data Capture, Vanderbilt University, TN, USA). For participants who consent and enrol using hard copy documents, data are transcribed into the portal by research team members, and patients (Gp1) are subsequently granted access to the portal via their personal electronic device for completion of questionnaires at later time points.

Access to the portal is enabled through SMS authentication (i.e., the user provides a code sent to their mobile phone via SMS to match the user to their electronic participant record). Following registration (at T_0_), participants complete a short pre-enrolment survey (designed on Qualtrics XM) to confirm they meet the study eligibility criteria. Following advice of a medical professional or senior researcher on site, the participant selects their respective cohort (i.e., tick-bitten patient (Gp1); patient presenting to ED or clinic due to a reason other than a tick bite (Gp2); or blood donor (Gp3) presenting at Lifeblood). Eligible participants are required to sign a consent form (counter-signed by the medical professional or researcher). Once the participant clicks ‘submit’, a copy of their signed consent form is automatically transferred to their record on *Monday.com* and copies of the patient information form and signed consent form are simultaneously emailed to the address provided by the participant. 

The next step in the electronic enrolment process consists of completing personal details. Through this process, personal information such as name, sex, date-of-birth, and postcode are collected. These identifiable details are stored on *Monday.com,* a Health Insurance Portability and Accountability Act (HIPAA)-compliant platform. In addition to being secure and separated from the other study databases, this software has advanced task management features required for efficient scheduling of follow-up appointments and other logistics tasks.

Following completion of personal details, the participant is directed to cohort-specific questionnaires online. Within the portal, Qualtrics XM, hosted on secure servers, is used for the development of customised, user-friendly structured surveys targeting each participant cohort (Gps 1–3) and time-point (T_0_–T_3_). Importantly, a de-identification code for each participant, generated by the study portal, is linked to their survey responses and to the participant’s *Monday.com* record. These unique codes are also used to anonymise biospecimens and results during downstream analyses. 

As noted previously, the portal is accessible by participants from their own electronic devices to complete questionnaires at T_1_–T_3_. In these cases, upon sign in, the participant’s mobile number is recognised by the system which will then link to the appropriate study questions. The study portal is notified by Qualtrics XM when the participant completes a questionnaire, and the portal in turn notifies Monday.com that the questionnaire has been completed.

Finally, all de-identified metadata captured in Qualtrics XM are automatically transferred into the project Master Database System hosted on REDCap, supported by secure servers. REDCap has broad capabilities to manage multisite, multi-cohort, longitudinal project data and is, therefore, chosen for long-term storage and management of metadata, clinical information, and laboratory results generated during the project.

Figure 2 summarises the role of each operating system integrating the portal, as well as the steps involved in the online enrolment process, follow-up visits, and data upload by researchers. 

### 4.5. Patient Withdrawal

Patients can withdraw from the study at any point by informing the project manager. Those patients who no longer want their samples stored and analysed will have their samples destroyed. Withdrawn patients complete a ‘Form for Withdrawal of Participation’, or in the event that the patient’s decision to withdraw is communicated verbally, the study doctor, senior researcher, or project manager will need to provide a description of the circumstances on the withdrawal form. Participants who are ‘lost to follow-up’ and do not return the project manager’s contact attempts are also considered withdrawn from the study. 

### 4.6. Management of Missing Data

A standard operating procedure (SOP) has been created for data quality control and management of missing data in REDCap. Missing data codes in the system are: ND (not done), UNK (unknown), and NA (not applicable). As a rule, ND must be used in cases where the participant did not answer a particular question and/or did not attend a scheduled sampling appointment. If researchers managing the data are unsure of why data are missing from a particular field, this could be clarified with patients during scheduled follow-up phone calls by the project manager. For instance, the code UNK will be used in situations where the project manager is informed by the participant that a particular question was not answered because information is unknown (e.g., patient does not recall how many occasions they had tick bites before). Lastly, NA is used in cases where a question is not applicable to that participant or cohort (e.g., about pregnancy to a male participant; and/or question about sample processing/storage times in cases where the participant did not attend the sampling appointment (ND)).

Another important strategy adopted is the standardised use of data collection status in REDCap, as outlined below:

- Unverified (yellow): All relevant data have been collected and recorded. However, this instrument contains fields with missing data codes such as “ND”. Researchers plan to follow-up with the participant to seek clarification or obtain further information from the patient;
- Complete (green): All participant records within that particular data collection instrument are up to date. Note that the instrument fields may contain some missing data codes such as ND; however, “complete” status indicates follow-up has been attempted or completed and no further action is required;
- Incomplete (red): This status is selected if, A) Participant decided to formally withdraw from the study; B) Participant could not be reached by phone or e-mail (i.e., loss to follow-up); C) Participant attended sampling appointment, however, did not consent to complete the survey.

In the data collection of instruments related to biospecimen processing/storage, a red code is used to show that the participant withdrew from the study and does not authorise the use of samples.

If required, additional details about each case will be recorded in an open text data collection instrument (“Patient log”) in REDCap.

### 4.7. Biospecimen Collection and Transport

#### 4.7.1. Gp1 and Gp2 (ED/Clinic Tick-Bitten Patients and Situational Controls)

Cohort-specific sampling kits are provided to participating medical centres before the start of each tick season (see example images in Figure S5). The “Tick Collection and Sampling Kit” (designed for Gp1 patients) contains a “Tick and Biopsy Collection Kit”, two “Blood Sampling Kits” (one for the day of enrolment and the other for one week later at T_1_), and information with instructions for medical personnel. The “Tick and Biopsy Collection Kit” is a ziplock bag containing sterile forceps for removing the tick, a punch biopsy tool, a suture kit, 5/0 nylon suture pack, a waterproof wound dressing, and pre-labelled sterile containers (70% ethanol and saline) for storage of the tick and biopsy, respectively. In cases where a second skin biopsy is collected, an additional “Biopsy Collection Kit” is included. 

The “T_0_ Blood Sampling Kit” consists of a colour-coded (red) lab mailer with relevant pathology request form(s), a vacutainer holder, needles (21G), and numbered blood collection tubes as follows: (1) PAXgene^®^ RNA vacutainer (2.5 mL) (Cat # 762165); (2) Lithium heparin vacutainer (LH) (2.0 mL) (Cat # 368494); (3–5) EDTA vacutainers (2.0 mL each) (Cat # 368841); (6) Serum separator vacutainer (SST) (8.5 mL) (Cat # 367958); and (7) SST (3.5 mL) (Cat # 367956). Kits used in regional areas do not contain a PAXgene^®^ tube because multi-omics analyses are time-critical and the turn-around times from regional centres cannot meet these deadlines. A colour-coded (green) “T_1_ Blood Sampling Kit” contains the same consumables as the corresponding T_0_ kit and are provided to the patient at the time of enrolment.

Medical personnel remove the frozen tick by grasping the tick’s mouth parts with forceps, close to skin level and slowly extract it from the skin until successfully detached. The tick is then placed in a sterile container containing 70% ethanol. Thereafter, if the anatomical site of a tick bite is deemed suitable and safe for a skin tissue biopsy, this procedure is performed following standard aseptic surgical procedure. A local anaesthetic (bupivacaine or lignocaine) is administered, and after 10 min, the biopsy is collected using the biopsy punch provided. The sample is placed immediately into a specimen jar containing sterile 0.9% saline irrigation solution and gauze.

Following the collection of the tick and biopsy, blood samples are collected either by the medical personnel or at the nearest participating pathology collection centre. A total volume of 22.5 mL of blood is drawn strictly in order as numbered on the tubes. The only exception where the blood sampling happens prior to skin biopsy collection is for sub-cohort Gp1 patients who are selected for spatial phenotyping studies (see “Spatial phenotyping” section). This is because inflammatory markers from the surgical procedure in the blood may preclude a reliable identification of tick bite-associated inflammatory markers. This variation of the standard protocol is not feasible within ED settings and, therefore, only occurs at selected clinics. 

From the day after enrolment and for 12 months, patients are contacted as necessary by the project manager to receive further instructions about their ongoing participation in the study. Additional colour-coded kits for T_2_ (yellow) and T_3_ (purple), containing the same selection of blood tubes as the T_0_ kit_,_ are posted to the Gp1 patient’s home approximately one week prior to their follow-up sampling appointment. 

In addition to Gp1, a “Blood Sampling Kit for Controls” and relevant instructions have also been designed for non-tick-bitten situational controls (Gp2). These kits contain the same blood collection materials provided for patients, except for a PAXgene^®^ tube. Gp2 controls have blood samples collected at the ED or are directed to the nearest participating pathology collection centre. 

All blood samples together with tick and biopsy containers (Gp1-T_0_ only) are placed back into the container and dispatched at room temperature (RT) to the pathology central laboratory using their courier network (Note: PAXgene^®^ tube must be stored at RT for 2 h before freezing at −80 °C, as per manufacturer’s recommendations). In case of sampling in regional areas (i.e., no PAXgene^®^ tube), samples are couriered chilled.

A complex logistics network has been established (via commercial agreements) with pathology collection centres throughout Australia for blood collection, transportation, routine testing and, in some cases, processing and the short-term storage of biospecimens. Site-specific request forms have been designed specifically for this study and are included in the appropriate kits. Details on partner pathology centres, associated courier destinations, and sample distribution strategies are summarised in Table 1.

#### 4.7.2. Gp 3 (Blood Donors)

A smaller volume of blood (total = 9.5 mL) is collected from consenting blood donors at the time of enrolment in compliance with the research ethics permit from Lifeblood. Therefore, a cohort-specific “Blood Sampling Kit for Donors” has been designed for this group and includes: a set of instructions, pathology request form, and numbered blood collection tubes as follows: (1) lithium heparin tube (2.0 mL); (2) and (3) EDTA tube (2.0 mL each); (4) SST (3.5 mL). Blood samples are collected by Lifeblood personnel at Donor Mobile Units and static Donor Centres. Tube 2 will be couriered to participating pathology centres for routine FBE (full blood examination). The remaining tubes are transported to Murdoch University (MU), if collected in WA, or the Australian Rickettsial Reference Laboratory (ARRL) (if collected in other states).

### 4.8. Biospecimen Management and Processing

Each biospecimen received at the laboratory for processing is aliquoted/transferred to de-identified colour-coded cryo-tubes. Labels display the unique participant code generated by the study portal as described previously, plus the date of birth, date of sample collection, biospecimen type, and time-point. Original PAXgene^®^ and the tick tube supplied in the kits are also de-identified prior to storage. 

At their intermediate and/or final destination, as indicated in Table 1 each biospecimen is processed within a Biosafety Cabinet Class II using aseptic technique, as per study SOP available at each participating laboratory. The sample processing methodology for samples not retained at pathology centres for routine testing (FBE and chemistries) is summarized below:

• Tick: Immediately after removal, the tick is placed in a pre-filled tube with 70% ethanol and stored at 4 °C for downstream analysis.
• Biopsy:
o Sectioning: Skin punch biopsies are longitudinally sectioned in the middle, approximately at the site of the tick bite, using a sterile surgical elongated triangular scalpel blade. Half of the skin (~2 mm) is placed in a labelled cryo-resistant tube and stored at −80 °C for metagenomic analysis; the other half is placed in 200µL of sterile phosphate-buffered saline (PBS), homogenised and inoculated into cell cultures (see “Microbial isolation” section).
o Preparation of biopsies for spatial phenotyping analysis (tick bite and control): For this sub-cohort, half of the skin biopsy collected from the patients (~2 mm) are placed in 10% of formalin for fixation in preparation for processing into a formalin-fixed paraffin-embedded (FFPE) format for sectioning prior to spatial analyses; the other half is placed in a labelled cryo-resistant tube and stored at −80 °C until required for metagenomic analysis.
• Blood samples:
o PAXgene® RNA blood: Whole blood is collected by venepuncture directly into RNA PAXgene® vacutainers prefilled with RNA stabilisation reagents. Immediately after collection, the PAXgene® tubes are inverted 10 times and stored at RT in an upright position for at least 2 h (and up to 4 h) before being stored in a −80 °C freezer.
o Lithium heparin blood: Whole blood is collected from participants in LH vacutainers. Upon arrival to the lab, the tube is centrifuged at 1000× *g* at RT for 10 min. Plasma (top layer) is collected (without aspirating red blood cells), placed into a cryovial labelled as “PLAS1”, and mixed by pipetting up and down 10 times. With a new tip, 200 µL of plasma is drawn from tube “PLAS1” and placed into tube “PLAS2”. This process is repeated by drawing up a second 200 µL of plasma from tube “PLAS1” which is then placed in tube “PLAS3”. All tubes (PLAS1-3) are stored immediately at −80 °C until required for proteomics, metabolomics, and cell-free circulating RNA analysis. The plasma-depleted cells that remain at the bottom of the lithium heparin vacutainer tube are stored in a cryovial at −80 °C for future epigenetic analysis.
o EDTA blood:
■ Whole blood EDTA samples: After venepuncture, the EDTA vacutainer is gently inverted to mix the blood, and three aliquots (200 µL and 2 × 400 µL) are transferred into cryovials and stored at −80 °C to be used for bacterial and protozoal profiling. An additional 500 µL of aliquot is made for viral metagenomic analysis.
■ EDTA blood smears: A total of 5 regular and 3 buffy coat blood smears are prepared using EDTA blood before and after centrifugation, respectively. The films are fixed in 100% methanol and stored in duly labelled, slide mailers.
■ Plasma EDTA samples: After aliquoting of whole blood, the EDTA tubes undergo an initial centrifugation at 1000× *g* for 15 min at RT. Subsequently, the top 90% of the plasma is slowly aspirated to a fresh 15 mL DNAse/RNase-free falcon tube without touching the buffy coat. An additional centrifugation of the plasma (2500× *g* for 15 min at RT) is then performed and the top layer of the plasma sample collected after the second centrifugation is stored in a cryovial at −80 °C for downstream cell-free RNA analysis.
■ Buffy coat samples: An additional EDTA blood tube is centrifuged at 5500× *g* for 5 min at RT. Thereafter, the buffy coat layer is collected and incubated in 5–8 mL of RBC lysis buffer (Cat # 158902) for 15 min at RT, followed by another centrifugation at 5500× *g* for 5 min. The supernatant is discarded, and the pellet is washed twice in PBS prior to resuspension in 600µL of PBS for immediate culture inoculation.
o SST blood: Blood collected in SST vacutainers is spun down at RT upon arrival to the lab at 2200× *g* for 15 min for serum separation. Approximately 1 mL of serum is retained for immediate serological testing. One serum aliquot of 500 µL is saved at −80 °C for viral analyses and six aliquots of 200 µL are stored at −80 °C for the study biobank at ARRL.

Upon receipt and processing of the samples, staff complete a site-specific Biospecimen Processing Form (BPF) (see Figure S6) with details such as time of arrival and centrifugations, volume of aliquots, colour and turbidity of samples, storage time, and any comments or deviation to protocol.

Following the procedures described above, selected sample aliquots according to the study operating procedure are distributed to a range of partner institutions for downstream analyses (further details provided in section: “Laboratory analyses: Collaborative generation of data”). Appropriate Material Transfer Agreements (MTAs) have been established between collaborating institutions handling the study samples and strict protocols are in place to monitor the transfer of samples between partner institutions. These include the completion and archiving of a Biospecimen Transfer Form (BPF) (Figure S7) with details on the origin, destination, and type/volume of samples transferred. All information recorded on BPFs and BTFs is subsequently recorded electronically in REDCap to ensure optimal tracking and standardization. 

### 4.9. Laboratory Analyses: Collaborative Generation of Data 

A combination of complementary traditional and cutting-edge laboratory and bioinformatics technologies are performed by a multi-disciplinary team at research institutions across Australia. These broad range of analyses were selected as a holistic approach to answer the research questions. Furthermore, the unique biobank collected during the present study provides an invaluable opportunity for multiple collaborative discoveries related to the aetiology, pathogenesis, and immune responses to Australian TBDs. Anonymised test results are stored in REDCap and then extracted to be analysed in conjunction with self-reported clinical, demographic, and psychometric data through an integrative statistical approach (as described in section “Statistical analyses and expected outcomes”). 

**Figure 3:**
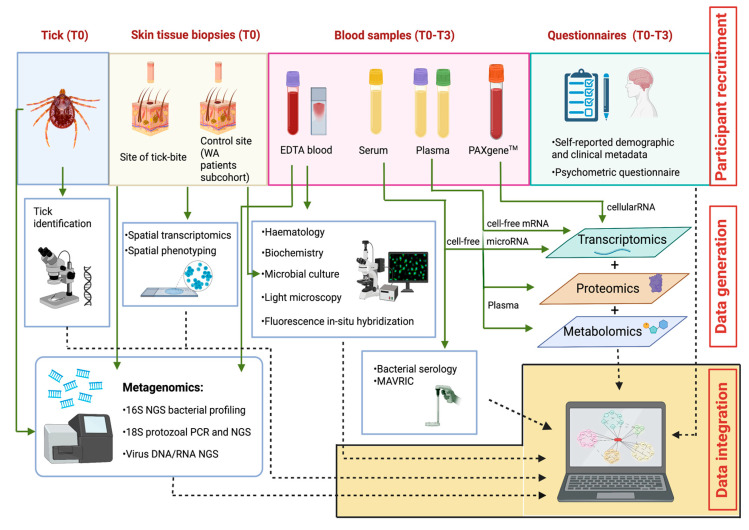
The Troublesome Ticks study: Overview of multi-disciplinary, multi-institutional laboratory analyses, and integration of resulting outputs. Image not drawn to scale. AB: Akoya Biosciences; ACDP: Australian Centre for Disease Preparedness; ARRL: Australian Rickettsial Reference Laboratory; GU: Griffith University; MoU: Monash University; MU: Murdoch University; PC: Pathology Centres; TKI: Telethon Kids Institute; UQ: University of Queensland.

The study strategies for the collaborative generation of data, including biological sampling, laboratory analyses, and study questionnaires, are depicted in Figure 3.

#### 4.9.1. Tick Identification

Whilst molecular technologies have been extensively applied for the characterisation of protozoal and microbial species, for ectoparasites (including ticks), morphological tools remain the gold standard for specimen identification. Limitations of morphological identification include the fact that morphological keys are not available for many Australian ticks. In addition, visualising certain features in damaged specimens or larvae/nymphs can be challenging. Therefore, recent studies have provided baseline molecular information for some Australian ticks to aid in phylogenetic reconstructions and taxonomic studies [52,53]. 

A combination of morphological and molecular tools is used to identify ticks in the present study. Firstly, ticks are visualised using an Olympus SZ61 stereomicroscope (Olympus) with an external Schott KL 1500 LED (Schott) light source. The instar, sex (if adult), and species of each tick specimen are identified using a combination of available morphological keys and species descriptions [23,54,55,56]. Molecular identification is performed as previously described [52], if ticks cannot be reliably identified using morphological tools alone.

#### 4.9.2. Microbial Isolation

Microbial isolation is an invaluable and irreplaceable technique for the identification of viable bacteria present in the sample. Moreover, in combination with molecular tools, it can increase detection sensitivity as it acts as a multiplier of microorganisms that may be present at low levels in human skin and blood. In vitro culture systems are also paramount for whole genome sequencing, development of diagnostic tests, antibiotic sensitivity tests, and future in vivo experiments to further elucidate pathogenesis mechanisms [57,58].

In the present study, attempts are made to culture potential bacterial pathogens from the buffy coat (extracted from EDTA blood) and half of the skin punch biopsy, in a range of cell lines that include ISE6 (tick), XTC-2 (amphibian), Ju56 (marsupial), and Vero (mammalian) [59,60]. Cultures are assessed weekly for up to 12 weeks by microscopy. At the end of the incubation period, the monolayer of each cell line is harvested and a portion of the supernatant and cells is stored until further testing for bacterial microorganisms using molecular methods (see section: “Molecular detection of tick-borne bacterial and protozoan pathogens”). Remaining cells are pelleted by centrifugation and cryopreserved in 10% dimethyl sulfoxide (DMSO) for further culturing of viral agents (see section: “Monoclonal antibodies to viral RNA intermediates in cells (MAVRIC)”).

#### 4.9.3. Microbial Serology

Serological tests for tick-borne pathogens known to be acquired in Australia (i.e., validated according to Australian conditions) are performed. Serum samples are tested by micro-immunofluorescence for antibodies against a panel of tick-borne rickettsial agents including *Rickettsia australis, R. honei, R. africae, R. rickettsiae, R. conori*, and *Coxiella burnetii*. In addition, given the exploratory nature of the present study, serological tests readily available are performed for other important rickettsial agents known to be flea-transmitted (*R. typhi* and *R. felis*), louse-transmitted (*R. prowazekii*), and mite-transmitted (*Orientia tsutsugamushi*) in Australia [61].

#### 4.9.4. Monoclonal Antibodies to Viral RNA Intermediates in Cells (MAVRIC)

Tick (ISE6—*I. scapularis*) or vertebrate (BSR—baby hamster kidney) cells lines are inoculated with cells retrieved from primary cultures of human tissue biopsies and buffy coat (see “Microbial isolation” section), and are incubated at the appropriate time and temperature. The supernatants are then harvested, and the cells fixed are in a solution of 4% formaldehyde with the addition of 0.5% Triton-X100 for 10 min at 4 °C [62,63]. A fixed-cell ELISA is then performed on plates using anti-dsRNA mAbs to detect long (>30 bp) double-stranded RNA (dsRNA), a molecule which is often present in RNA virus-infected cells as either the genomic form (e.g., Reoviruses) or as a replicative intermediate (e.g., flaviviruses) [64]. The recognition of dsRNA by monoclonal antibodies to viral RNA intermediates in cells (MAVRIC) is sequence-independent and, because long dsRNA is not found in uninfected cells, allows for the detection of RNA viruses from a diverse range of viral families [64].

#### 4.9.5. Haematology and Biochemistry

Routine pathology testing is performed at pathology collection centres. Haematology (i.e., FBE) is analysed for every patient at all time-points and for Gp2-controls at T_0_. Routine clinical chemistries including electrolytes, urea, and creatinine (EUC); liver function test (LFT) and C-reactive protein (CRP) are performed at all time points for Gp1 tick-bitten patients and once in both external controls (Gp2 and Gp3). 

#### 4.9.6. DNA and RNA Extractions from Ticks, Tissue, Blood, and Cultured Cells

Dual DNA and RNA extraction methods are performed on ticks and skin punch biopsies using the Qiagen DNeasy Blood & Tissue kit and QIAamp DNA mini kit (Qiagen, Germany), respectively, as per previously described [30,32]. An aliquot of each sample resulting from dual extractions, which are dedicated to virus detection analysis, undergo DNAse treatment. This is to avoid carryover DNA that might negatively impact library preparation for next-generation sequencing (NGS).

DNA is isolated from whole blood using the QIAamp DNA Microbiome Kit (Qiagen), through an automated DNA extraction platform. This kit involves the effective depletion of host DNA, and therefore, maximizes bacterial DNA coverage in 16S rDNA (16S)-based NGS. 

For both 16S and 18S rDNA (18S; for eukaryotic parasites)-based analyses, DNA samples are obtained from cell cultures and an additional aliquot of whole blood from each participant using the QIAamp DNA mini kit. For virus detection analyses, RNA extractions from blood samples and cultured cells are performed using a QIAamp RNA mini kit (Qiagen), according to the manufacturer’s recommendations.

#### 4.9.7. Molecular Detection of Tick-Borne Bacterial and Protozoan Pathogens

NGS is used to amplify the V1-2 hypervariable region of the bacterial 16S rRNA gene in skin tissue biopsies (T_0_), ticks (T_0_), and blood samples from patients (T_0_–T_3_) as well as internal and external controls (T_0_), using established pipelines [32]. NGS identifies communities of microorganisms present in a sample including known or novel organisms belonging to ‘taxa of interest’, i.e., tick-associated pathogenic and endosymbiotic organisms [19,65]. In this research, ‘taxa of interest’ are defined as genera within spirochaetes, alphaproteobacteria and gammaproteobacteria, specifically; *Anaplasma*, *Bartonella*, *Borrelia*, *Coxiella*, *Ehrlichia*, *Francisella*, *Midichloria*, *Neoehrlichia*, *Rickettsia*, and *Rickettsiella* [1,66,67,68]. Importantly, the assay identifies the presence of any putative human tick-borne pathogens previously detected in Australia, including novel *Borrelia* spp., *Anaplasma* spp., *Ehrlichia* spp., ‘*Candidatus* Neoehrlichia’ spp., *Mycoplasma* spp., and *Francisella* spp. [19,20,21,27,32,69,70].

Conventional PCR (cPCR) and Sanger sequencing of longer amplicons are also used to further characterise novel potential pathogens identified. Additionally, PacBio Sequel II may be used on selected samples to sequence the 16S-ITS-23S rRNA operon, for more robust phylogenetic reconstructions [71].

In addition to 16S bacterial profiling, ticks, skin tissue biopsies, and blood samples are screened using metabarcoding and nested PCR assays targeting eukaryotic 18S regions [72,73]. In addition, genus-specific cPCR and Sanger sequencing are conducted on skin biopsies and blood samples, and subsequently on corresponding ticks to any positive human specimen. These methods target longer 18S amplicons from three key genera of tick-associated protozoan pathogens known to occur in Australian native wildlife and ticks: *Trypanosoma*, *Babesia*, and *Theileria* [30,74,75,76,77,78,79,80,81]. Based on the results, *Trypanosoma*-specific 18S metabarcoding [30,33] may be required to investigate potential human mixed infections with trypanosomes. Finally, Sanger sequencing of additional loci are performed for the further characterisation of any eukaryotic pathogen detected in human samples (e.g., [76,77]). 

#### 4.9.8. Next-Generation Sequencing for RNA Virus Detection

Purified RNA samples from ticks, skin tissue biopsies, and blood are subjected to a whole transcriptome pipeline; TruSeq Stranded mRNA Total Library Prep with Ribo-Zero Gold rRNA depletion and unique dual indexing, sequencing on the Illumina NovaSeq 6000, 100 bp paired-End, 50 M read pairs. To identify novel and known viruses, sequences undergo a virus discovery bioinformatics pipeline as previously described [82]. A computational pipeline is used to obtain coverage statistics and to validate potential misassemblies. Lastly, maximum-likelihood phylogenetic trees are produced for the phylogenetic placement of novel viruses within respective families [82].

#### 4.9.9. Light Microscopy and Fluorescence In Situ Hybridization (FISH)

Blood films from participants who tested positive for bacterial and/or protozoan pathogens by molecular assays are screened by light microscopy and fluorescence in situ hybridization (FISH). Briefly, thin whole blood and buffy coat smears are stained using Modified Wright’s stain. A cover-slip is placed over the stained blood smear and the preparation examined by light microscopy at 200×, 400×, and 1000× magnification for the presence of microorganism(s) detected by NGS and PCR. Images are recorded using an Olympus DP71 Advance digital camera. For FISH analysis, a genus-specific DNA probe with a 5′ 6-carboxyfluorescein fluorescent tag at the 5′ end (IDT Australia) is manufactured and applied as per previously published protocols [69,83]. 

#### 4.9.10. Transcriptomics

The use of high-dimensional multi-variate analyses which comprises genomics, transcriptomics, proteomics, and metabolomics methodologies (collectively referred to as “multi-omics”) is a useful approach to study the pathophysiology of disease, identify biomarkers and generate insights into possible interventions, and has been successfully applied to vector-borne disease research in other parts of the world [36]. Transcriptomics can specifically lead to a better understanding of the current phenotype of cells, which, differently from genomics, can fluctuate due to prevalent cellular stimuli. When challenged with a tick bite and pathogen transmission, for example, the immune transcriptomic expression of humans can provide insight into pathogenesis and symptomology [36].

Cellular total RNA transcriptomics analyses are conducted in longitudinal RNA samples (at T_0_, T_1_, T_2_, and T_3_) extracted from whole blood PAXgene^®^ samples using the dedicated PAXgene^®^ Blood miRNA Kit. The extracted cellular RNA is put through quality control steps, such as NanoDrop, bioanalyzer and Qubit, before sequencing. Cell-free circulating RNA is obtained via the extraction of RNA from EDTA plasma using the Qiagen miRNeasy serum/plasma advanced kit or the Norgen plasma/serum RNA purification mini kit. The extracted cell-free RNA goes through quality control steps, such as genomic DNA contamination qPCR, bioanalyzer, and Qubit, before being sequenced with the Takara RNA sequencing library [84].

MicroRNAs are isolated from 200 μL of human serum using the miRNeasy micro kit (Qiagen) as per the manufacturer’s instructions. In the event that plasma samples are originally obtained using sodium heparin vacutainers, the eluted RNA is treated with 1U heparinase I (Sigma Aldrich, H2519) at 25 °C for 30 min to remove any remaining heparin. Complementary DNA libraries are generated using the QIAseq miRNA Library Kit and QIAseq miRNA NGS 48 Index IL (Qiagen) as per the manufacturer’s protocol. All libraries are assessed for quality control on the Bioanalyser 2100 using the High Sensitivity DNA Kit (Agilent) to ensure a correct insert size and minimal adapter or primer carryover. Libraries are subjected to 100 bp single end sequencing on the NovaSeq 6000 (Illumina). The miRDeep2 quantifier [85] is used to map and quantify reads against the latest miRBase human reference (version 22) [86]. 

#### 4.9.11. Spatial Phenotyping

Spatial phenotyping is used in the present study to identify transcriptional pathways that perturbed the normal physiology of the skin after a tick bite. It will help elucidate the identity and amount of cellular infiltrate on a small portion of tissue, in a greater magnitude of multiplexing compared to commonly used immunohistochemistry [87].

The FFPE skin samples are sectioned transversely (with each section containing both the epidermis and dermis) and multiple sections are mounted on a single microscope slide. To gain orientation and provide a morphology guide for the later selection of regions of interest, a haematoxylin and eosin (H&E) stain is also performed. A set of morphology markers (PanCK, CD48, CD4/8, and CD68) are used to stain the sections in order to, respectively, identify epithelial cells, immune cells, T cells, and macrophages. These markers will further inform the downstream untargeted full transcriptomics analysis that targets over 18,000 protein coding genes, spatially on each slide [87]. The analysis of the spatial genomics data is performed using the NanoString’s GeoMx Digital Spatial Profiling platform and correlated with the cellular and cell-free transcriptomics data [87].

#### 4.9.12. Plasma Proteomics Analysis

Proteomics analysis is used to identify and quantify expressed proteins and match protein sequences to the corresponding molecular function, which can be used as reliable biomarkers to describe the pathogenesis of diseases [36].

In the present study, plasma proteomics analysis is conducted by SomaLogic Inc. (Boulder, CO, USA) using SOMAscan technology, which is an aptamer-based proteomics assay capable of measuring 1305 human protein analytes in serum, plasma, and other biological matrices with high sensitivity and specificity. Normalized and non-normalized Somascan data are obtained and analysed using pre-defined standard analytic pipelines [88]. 

#### 4.9.13. Plasma Metabolomics Analysis

Metabolomics data provide insight into small molecules that are intermediates or products of metabolic pathways, which have increasingly been associated with immunological response pathways. Together with transcriptomics and proteomics, analyses of metabolites also enable the identification of biomarkers for TBDs [36].

Plasma metabolomics is analysed on plasma samples collected from EDTA blood samples using Metabolon’s HD4 platform LC-MS/MS for the identification of global plasma metabolites. The metabolites will be selected based on the retention index (RI), mass-matched to a reference library and MS/MS forward and reverse score for biochemical identification [88].

#### 4.9.14. Whole Blood Epigenetics Analysis

Epigenetics is adopted to study molecules and mechanisms (e.g., histone modifications, DNA methylation, and noncoding RNAs) that can generate gene activity states that differ from a given DNA sequence. This methodology has been previously applied on research of tick biology and vectorial capacity [89] as well as human immune responses to important diseases such as Lyme disease overseas [90].

In the present research, plasma-depleted blood is used for genomic DNA (gDNA) extraction using DNeasy Blood & Tissue Kit (Qiagen) following the manufacturer’s instructions. gDNA samples are then subjected to overnight bisulfite conversions with the EZ DNA Methylation kit (ZymoResearch, Irvine, CA, USA), and 160 ng of the bisulfite-converted DNA (bcDNA) samples is used to perform global DNA methylation profiling on the Illumina MethylationEPIC beadchips. Using the Illumina GenomeStudio software package, average beta values are calculated, and the DNA methylation data quality is assessed [84].

#### 4.9.15. Clinical and Demographics Metadata

Metadata and clinical data captured through questionnaires include:

1. Gp1-T_0_: sex, age, postcode, details about the present or recent tick bite (e.g., body site, geographic location, approximate duration of tick attachment), tick-killing method (if not still attached), local and generalised symptoms (if any), overseas travel history in the past 6 months, history of tick bites in Australia and overseas and any associated symptoms, health history including any previous diagnosis of neurological, cardiac and mental disorders, previous diagnosis of MMA, and current prescribed medication(s);
2. Gp1-T_1–3_: current (persistent and new) local and generalized symptoms, any new specific diagnosis (e.g., COVID-19), current prescribed medication(s); and
3. Gp2 and Gp3: sex, age, postcode, history of tick bites in Australia and overseas, associated symptoms, health history, and current prescribed medication(s).

#### 4.9.16. Psychometric Analyses and Personality Profiling

This study will investigate psychological correlates of patient responses to tick bites, with and without subsequent illness, to ascertain whether there is a relationship between the baseline psychological profile and the development of particular symptoms after a tick bite (e.g., fatigue, anxiety, depression, headaches). To this end, three standardised self-administered psychometric measures will be conducted on patients and controls to measure stress, anxiety, emotional reactivity, depression, and somatisation. Short forms of the Depression, Anxiety and Stress Scales (DASS-21), Health Anxiety Inventory (SHAI) and Cognitive and Behavioural Responses Questionnaire (CBRQ) will be administered at T_0_ (Gp1, Gp2, and Gp3) and T3 (Gp1). DASS-21 will also be administered to tick-bitten patients (Gp1) at T_1_ and T_2_.

In addition to the measures mentioned above, the NEO-FFI-3 (i.e., short version of the NEO Five-Factor Inventory) personality questionnaire will be administered once in Gp1, and in external blood donor controls (Gp3), to investigate whether an individual’s personality influences symptoms after a tick bite.

### 4.10. Statistical Analyses

It is expected that most patients with tick attachment will not have symptoms on presentation. Some patients will progress to develop local or systemic symptoms, but the number of patients with a tick bite that will do so is uncertain. However, based on limited tick-bite illness data overseas [46] and clinical observations following *I. holocyclus* bites at the Sydney hospitals, we estimate that approximately 10% of patients with a tick bite will develop local or systemic symptoms consistent with this case definition for acute or chronic illness; therefore, the total number of symptomatic tick bite cases (Gp 1A) is expected to be 90. Due to public interest in tick-associated illness, a high compliance rate among patients is expected. Overall, we expect to have follow-up data in 90% (i.e., a 10% drop-out rate) of cases (final total of 81 cases in group 1A). We anticipate a higher drop-out rate (20%) from asymptomatic internal controls. There will be no drop-out in external controls as these will be tested only once. 

Sample size and power calculations have been performed based on a Fisher’s exact test for comparison of proportions and indicate that at 0.05 significance, 81 participants are required in each group for an 80% chance of capturing ‘small to moderate’ interactions between each of the fixed effects and the multinomial dependent variable. Correction for multiple hypothesis testing will not be performed. Statistical packages in R Studio will be used for all analyses in R Studio [91].

Case (i.e., Gp1A—symptomatic tick-bitten patients) data will be compared with three comparison groups; tick-bitten patients that do not develop symptoms (nested case–controls Gp1B) and non-tick-bitten controls (Gp2, ED controls; and Gp3, blood donors) to test the primary hypothesis that that laboratory abnormalities and psychological responses are more common in symptomatic tick-bitten individuals than in non-symptomatic patients with a history of tick bites or population controls.

The presence or absence of microbes in skin and blood samples and clinical chemistries, immune profiles, and serological data, will be compared between cases (Gp1A) and the three control groups (Gp1B, Gp2, and Gp3). Case definition(s) will be further refined through the definition of one or more syndromes based on the presence of clinical, psychometric, biochemical, immunological, and serological markers using Classification and Regression Trees (CART) analysis [92].

Statistical comparisons between microbial populations in ticks, tissues and blood samples of cases, and the controls (Gp1B, Gp2, and Gp3) will provide new information about microbes transmitted to people during a tick bite. NGS bacterial profiling results will be represented as a stacked bar plot of bacterial composition. In addition, principal coordinate analysis (PCoA) on weighted UniFrac dissimilarity measurements will be used to compare the microbial composition between sample types (e.g., tissue vs. blood vs. tick); and cases (Gp1A) vs. controls (Gp1B, Gp2, and Gp3).

Metagenomics results will be integrated with data outputs from transcriptomics, proteomics, and metabolomics (comparing cases vs. three control groups) using a Data Integration Analysis for Biomarker discovery using Latent cOmponents (DIABLO) [93]. This will allow for distinctions between potential biomarkers of tick bites and other illness not linked to tick bites, as well as to identify biomarkers associated with tick-transmitted microorganisms or non-infectious antigens. For example, external controls (Gp2 and Gp3) will be particularly useful if samples are positive for microbes in both cohorts of the tick-bitten group and would provide evidence of a subclinical exposure to tick organisms in Gp1B.

Differences between microbes identified, immunological and pathology markers, psychometric profiles, clinical symptoms, and metadata in symptomatic tick-bitten individuals (Gp1A) and controls (Gp1B, Gp2, and Gp3) will be determined using Fisher’s exact tests (for dichotomous exposures) and t-tests (for normally distributed continuous exposures, following transformation as required). A Generalised Linear Mixed Model (GLMM) will be used to test whether there is a relationship between a psychological profile and symptoms after a tick bite. 

## 5. Expected Outcomes

It is anticipated that this research will fill important knowledge gaps about the aetiology, epidemiology, and pathophysiology of tick-associated disease in Australia, which will ultimately inform the development of improved diagnostic, management, and treatment protocols for patients bitten by ticks. Specific outcomes we expect to achieve in this study are outlined below.

1. Through nationwide patient recruitment, data on the incidence and geographical distribution of tick-associated illness will be generated;
2. Clinical information and psychometric profiling will provide a better understanding of the physical and psychological determinants, impacts of tick-associated illness, and the development of DSCATT on patients;
3. Investigation of both infectious and non-infectious aetiologies using a holistic multi-omics approach, associated with clinical data, chemistries, and haematological analysis in patients and controls will give insight into the aetiology and pathophysiology of tick-associated illness;
4. Molecular testing of blood samples, skin biopsies, and ticks will determine if the transmission of microorganisms is a factor, and the comparison of patients and controls will bring rigour to addressing Koch’s postulates [94] if infection is associated with tick bites; it will also identify links between microorganisms and tick species leading to the knowledge of potential vectors;
5. Microbial culture of skin biopsies and blood samples will permit the development of serological assays if tick-associated illness and DSCATT are associated with infectious disease;
6. The development of a valuable specimen bank with extensive associated archived clinical data that will be available to researchers in order to evaluate diagnostics tests and treatment efficacy well into the future.

## 6. Discussion

The Troublesome Ticks study protocol consists of a multi-disciplinary holistic strategy to investigate the aetiology of tick-associated illness in Australia. The experimental design, selected technology platforms, and robust databases generated enable a clearer definition of epidemiological, clinical, and pathological features of acute and long-term sequelae post-tick bite. 

The sample collection commenced in August 2020 and will be completed by March 2023. Unfortunately, despite great efforts to promote the project, COVID-19 restrictions significantly impacted our ability to recruit participants and medical practices during the first two tick seasons of the project (2020/2021 and 2021/2022). Movement restrictions dictated by local governments at various times since the start of the pandemic precluded visits to EDs, clinics, or pathology collection centres. It is one of our current priorities to expand the range of patient recruitment activities ahead of the next tick season (2022/2023) when we expect significantly higher enrolment numbers. A potential limitation associated with difficulties to advertise the project broadly might be an underrepresentation of TBDs in regional areas. If that is the case, this limitation will be duly addressed in publications reporting outcomes of this research. 

Face-to-face enrolment of Gp3 blood donors was also prevented due to COVID restrictions; however, an online recruitment system was developed and samples from the first cohort of consenting donors in NSW were successfully collected in 2021. It is expected that in-person recruitment will resume in 2022, for which logistic planning is underway. For the donors who have already participated, the blood samples were collected at their next donation at an identified site (Dee Why Donor Mobile Unit) and not at the same time as they consented and enrolled into the study. Donors were encouraged to complete the study questionnaires within 1 week of their donation, and thus, the sample collection; however, this was not always possible.

The researchers also acknowledge that potential psychological impacts of the coronavirus pandemic may introduce bias to the psychometric measures, albeit such impacts are likely to be similar in both patients and controls. In addition, these may not impact the assessment of more stable personality traits. Limitations associated with this important component of the study will be duly addressed by our clinical psychologist and statistician during the data analysis stage.

Despite the constraints reported above, we believe that our study protocol, underpinned by prospectively designed data collection, recruitment of three matched control groups, and sophisticated integrative data analysis, will generate the most comprehensive knowledge on human TBD in Australia to date. Our team is prepared to tackle limitations inherent to a potentially smaller sample size than that originally planned. Importantly, the approach to integrated multi-omics analysis will allow for significant associations to be established using relatively smaller sample sizes, based on the integration of multiple pathways (producing ~500,000 assay variables per sample). It is, therefore, anticipated that links between specific infectious and non-infectious aetiologies and patients who experience longer-term symptoms will be identified, for which diagnostic and treatment protocols can be safely developed.

It is anticipated that the research plan described here will serve as a proxy for upcoming research aiming to investigate the aetiology, clinical outcomes, immune and psychological responses associated with human tick-bites in other parts of the globe. It is important, however, that key factors are considered and adjustments are made (if possible) for this protocol to be applicable to other locations other than Australia. For instance, geographic, socio-economic, and cultural aspects would likely influence the effectiveness of project advertisement and participant recruitment. In addition, relatively easy access to laboratories and efficient logistics for sample collection and transportation must be carefully planned to ensure the suitability of biospecimens collected for each laboratory analysis proposed.

## Figures and Tables

**Table 1:** Nation-wide couriering of specimens collected as part of the Troublesome Ticks project (Gp1—tick-bitten patients and Gp2—ED controls).

Sample Collection Location	Biospecimen	Intermediate Destination (Via Pathology Centre (PC) Courier Network)	Final Destination (Via Commercial Courier Services)
Sydney	Tick *	PC central lab (Storage at −4 °C)	Batch shipment to MU at the end of each tick season.
	Skin biopsy *	PC central lab	Immediate onward shipment to ARRL (half biopsy tested at ARRL and half frozen at −80 °C until batch shipped to MU).
	Blood tubes: 1 (PAXgene^®^ *^,#^) and 2 (LH)	PC central lab (Heparinised plasma harvested and stored with PAXgene^®^ *^,#^ at −80 °C within 4 h of collection)	Batch courier to MU at the end of each tick season.
	Blood tubes: 4 (EDTA), 5 (EDTA) and 7 (SST)	PC central lab (SST centrifugation)	Immediate onward shipment to ARRL (Tubes 4 and 7 used for analyses at ARRL, tube 5 frozen at −80 °C or until batch couriered to MU).
	Blood tubes: 3 (EDTA) and 6 (SST)	PC central lab (FBE, chemistries, blood smears, EDTA plasma harvesting within 4 h of sample collection)	Batch courier of blood smears and frozen plasma to MU at the end of each tick season.
Western Australia	Tick *	PC central lab (SST centrifugation)	Immediate pick-up by a MU researcher (Storage at −4 °C at MU).
	Skin biopsy *^,^^		Immediate pick-up by a MU researcher (half frozen at −80 °C at MU and half express couriered to ARRL).
	Blood tubes: 1 (PAXgene^®^ *^,#^), 2 (LH), 4 (EDTA), 5 (EDTA) and 7 (SST)		Immediate pick-up by a MU researcher (express shipment of tubes 4 and 7 to ARRL; remaining samples processed, aliquoted and stored at −80 °C at MU).
	Blood tubes 3 (EDTA) and 6 (SST)	PC central lab (FBE and chemistries)	N/A
Other locations	Tick *	PC central lab (SST centrifugation)	Immediate onward shipment to ARRL, then batch shipment to MU after each tick season (Storage at −4 °C).
	Skin biopsy *		Immediate onward shipment to ARRL (half biopsy tested at ARRL and half frozen at −80 °C for until batch shipped to MU).
	Blood tubes 1 (LH), 3 (EDTA), 4 (EDTA) and 6 (SST)		Immediate onward shipment to ARRL (Heparinised plasma from tube 1, and tube 3 stored at −80 °C until batch shipped to MU. Tubes 4 and 7 used for analyses at ARRL).
	Blood tubes 2 (EDTA) and 5 (SST)	PC central lab (FBE and chemistries)	N/A

ARRL: Australian Rickettsial Reference Laboratory; EDTA: Ethylenediaminetetraacetic acid; FBE: full blood examination; LH: Lithium heparin; MU: Murdoch University; N/A: Not applicable; SST: Serum-separating tube. * Gp1-tick-bitten patients only; ^#^ Perth metro areas only; ^ If double biopsy (bite site and control), a MU researcher will be on site and take these samples to Telethon Kids Institute (TKI) for immediate processing.

## Data Availability

Data sharing is not applicable to this article as no datasets were generated or analysed. All relevant materials are published in the article and its supplementary files.

## Supplementary Materials

The following supporting information can be downloaded at: https://www.mdpi.com/article/10.3390/pathogens11111290/s1, Figure S1: Troublesome Ticks research: participant recruitment poster; Figure S2: Troublesome Ticks research: participant recruitment flyer, Figure S3: Troublesome Ticks research: blood donor recruitment flyer, Figure S4: Troublesome Ticks research online portal landing page, Figure S5: Troublesome Ticks research sampling kits design, Figure S6: Troublesome Ticks research-Biospecimen Processing Form, Figure S7: Troublesome Ticks research-Biospecimen Transfer Form, Video S1: Troublesome Ticks research: participant recruitment, Video S2: Troublesome Ticks research: medical staff recruitment, Video S3: Troublesome Ticks research protocol for medical staff.

## Abbreviations

The following abbreviations are used in this manuscript:

ACDP	Australian Centre for Disease Preparedness
AGRF	Australian Genome Research Facility
ARRL	Australian Rickettsial Reference Laboratory
ASCIA	Australasian Society of Clinical Immunology and Allergy
BPF	Biospecimen Processing Form
BTF	Biospecimen Transfer Form
CART	Classification and Regression Trees
CBRQ	Cognitive and Behavioural Responses Questionnaire
CFS	Chronic fatigue syndrome
CRP	C-reactive protein
DASS-21	Depression, Anxiety, and Stress Scales
DIABLO	Data Integration Analysis for Biomarker discovery using Latent cOmponents
DSCATT	Debilitating Symptom Complexes Attributed to Ticks
ED	Emergency department
EUC	Electrolytes, urea, and creatinine
FFPE	Formalin-fixed paraffin-embedded
FBE	Full blood examination
FISH	Fluorescence in situ hybridization
GLMM	Generalised Linear Mixed Model
GP	General practitioner
Gp	Group
HIPAA	Health Insurance Portability and Accountability Act
LD	Lyme disease
LFT	Liver function test
LH	Lithium heparin
MAVRIC	Monoclonal antibodies to viral RNA intermediates in cells
ME	Myalgic encephalomyelitis
MMA	Mammalian meat allergy
MTA	Material Transfer Agreement
MU	Murdoch University
NA	Not applicable
ND	Not done
NEO-FFI	NEO Five-Factor Inventory
NGS	Next-generation sequencing
NSW	New South Wales
PBS	Phosphate-Buffered Saline
PC	Pathology Centre
PCoA	Principal coordinate analysis
Qld	Queensland
REDCap	Research Electronic Data Capture
SHAI	Health Anxiety Inventory
SOP	Standard Operational Procedures
SST	Serum-separating tube
TBDs	Tick-borne diseases
TKI	Telethon Kids Institute
UNK	Unknown
UQ	The University of Queensland
WA	Western Australia
