# Supplementary material for: The Troublesome Ticks Research Protocol: Developing a Comprehensive, Multidiscipline Research Plan for Investigating Human Tick-Associated Disease in Australia
Source: Pathogens. 2022 Nov 3;11(11):1290. doi: 10.3390/pathogens11111290 (PMC9694322; doi:10.3390/pathogens11111290)
Supplement: Supplementary file 1 [file pathogens-11-01290-s001.zip › Additional files/Figure S7.Biospecimen Transfer Form.pdf]

Troublesome Ticks study

Biospecimen Transfer Form

Murdoch University

PLEASE COMPLETE THIS FORM BEFORE SENDING OR UPON RECEIVAL OF BIOSPECIMENS

PARTICIPANT ID		
VISIT (if known)		I__I T0 I__I T1 I__I T2 I__I T3
Date of sample collection		_____/_____/______
ORIGIN		I__I MU I__I ARRL I__I NSWHP<br>I__I UQ I__I TKI I__I CSIRO
DESTINATION		I__I MU I__I ARRL I__I NSWHP<br>I__I UQ I__I TKI I__I CSIRO
SPECIMENS BEING DISPATCHED<br>(Specify number/volume of each biospecimen)		I____I Tick I____I Biopsy I____I PAXgene<br>I____I Heparin I____I EDTA I____I SST<br>Plasma aliquots: Number I__I Volume each I___I<br>Serum aliquots: Number I__I Volume each I___I<br>Number I__I Volume each I___I
ATTACHED COPY OF COMPLETED "BIOSPECIMEN PROCESSING FORM"?		I__I YES I__I NO I__I NA
RESEARCH STAFF NAME AND SIGNATURE	Sample dispatch	Date: / /
	Sample receival	Date: / /
COMMENTS		

<u>Sender:</u> Please send a signed copy of this form to destination lab along with complete "Biospecimen processing form" and samples. Please also archive a copy in your laboratory.

<u>Recipient:</u> Please sign this form and archive a copy in your laboratory.

<u>MU and ARRL Researchers ONLY: Record SAMPLE TRANSFER details on REDCap</u>
