# Supplementary material for: The Troublesome Ticks Research Protocol: Developing a Comprehensive, Multidiscipline Research Plan for Investigating Human Tick-Associated Disease in Australia
Source: Pathogens. 2022 Nov 3;11(11):1290. doi: 10.3390/pathogens11111290 (PMC9694322; doi:10.3390/pathogens11111290)
Supplement: Supplementary file 1 [file pathogens-11-01290-s001.zip › Additional files/Figure S3.Blood donor recruitment flyer.pdf]

How do I avoid tick bites?

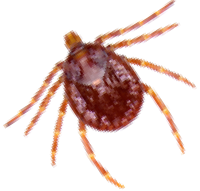

In gardens by:

- Avoiding overgrown or dense foliage and areas of deep leaf litter
- Keeping lawns mown, removing long grass from garden borders
- Regular trimming of bushes and shrubs and removal of low branches
- Removing leaf and bark litter
- Removing rubbish which could provide ticks with shelter
- Ensuring these 'tick-reduced' areas are maintained in locations where children and pets are likely to access regularly

Outdoors:

- Wearing suitable clothing that reduces the amount of bare skin that is exposed
- Tucking long pants into socks and wear light coloured clothes so that any crawling ticks can be more easily seen and removed
- Using commercially available insect/arthropod repellents

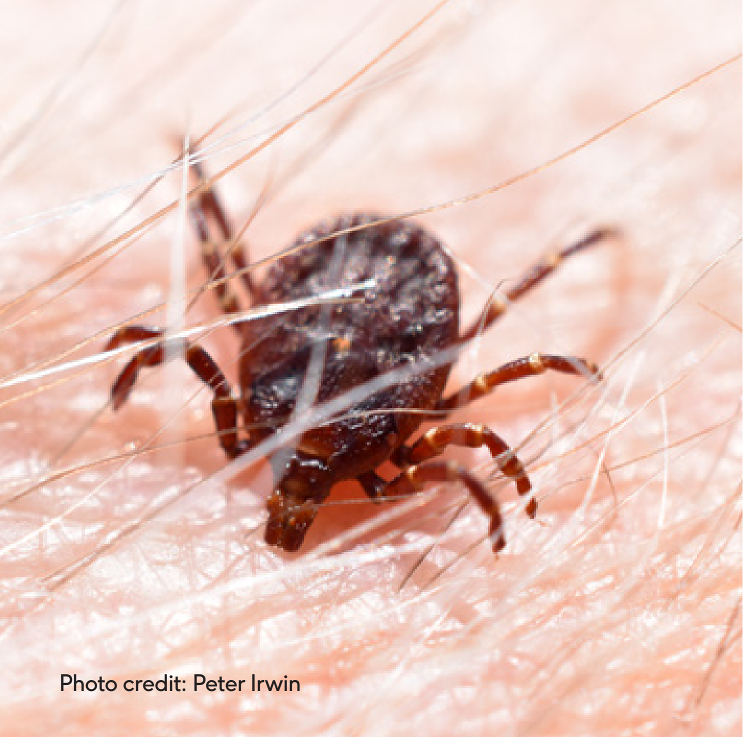

Photo credit: Peter Irwin

Murdoch UNIVERSITY

For more information, please contact our Project Manager on 1300 81 70 70, email DSCATT@rickettsialab.org.au or visit our website: tickstudy.murdoch.edu.au

This study has been approved by the Murdoch University Human Research Ethics Committee (permit 2019/124), the Northern Sydney Local Health District (permit 2019_ETH12032) and the ARC Lifeblood (permit 2019-20)

CRICOS Provider Code 00125J MCO0004789
Cover photo credit: Stephen Doggett

Research partners

NHMRC

Northern Beaches Hospital

Australian Rickettsial Reference Laboratory

MONASH University

THE UNIVERSITY OF WESTERN AUSTRALIA

THE UNIVERSITY OF QUEENSLAND AUSTRALIA

Australian Red Cross Lifeblood

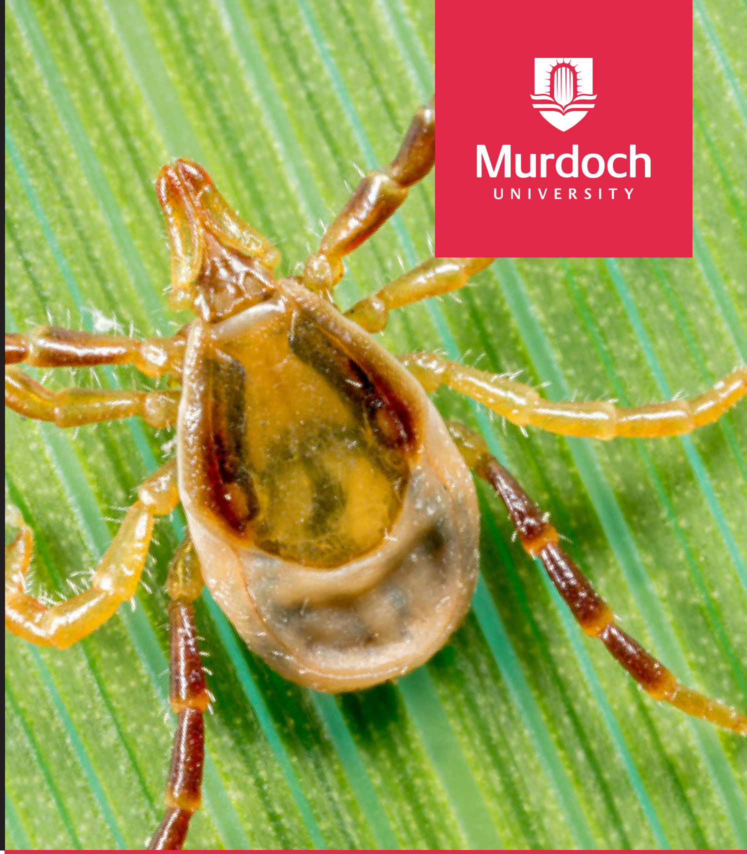

Murdoch UNIVERSITY

Participants required for research project on tick bites in Australia.

Please consider participating in this ground-breaking medical research to help determine the cause(s) of Debilitating Symptom Complexes Attributed to Ticks (DSCATT) in Australia.

Phone 1300 81 70 70

Why This Study?

In Australia many people get bitten by ticks every year. There is uncertainty about why and how ticks make people unwell in Australia, yet there are now thousands of patients suffering from fatigue, arthritis, chronic pain, headaches and psychological symptoms following a tick bite. These illnesses are known as 'Debilitating Symptom Complexes Attributed to Ticks' (DSCATT).

This study will provide important insights into the symptoms of DSCATT and help find ways to diagnose and treat the disease.

The project is funded by the National Health and Medical Research Council (NHMRC).

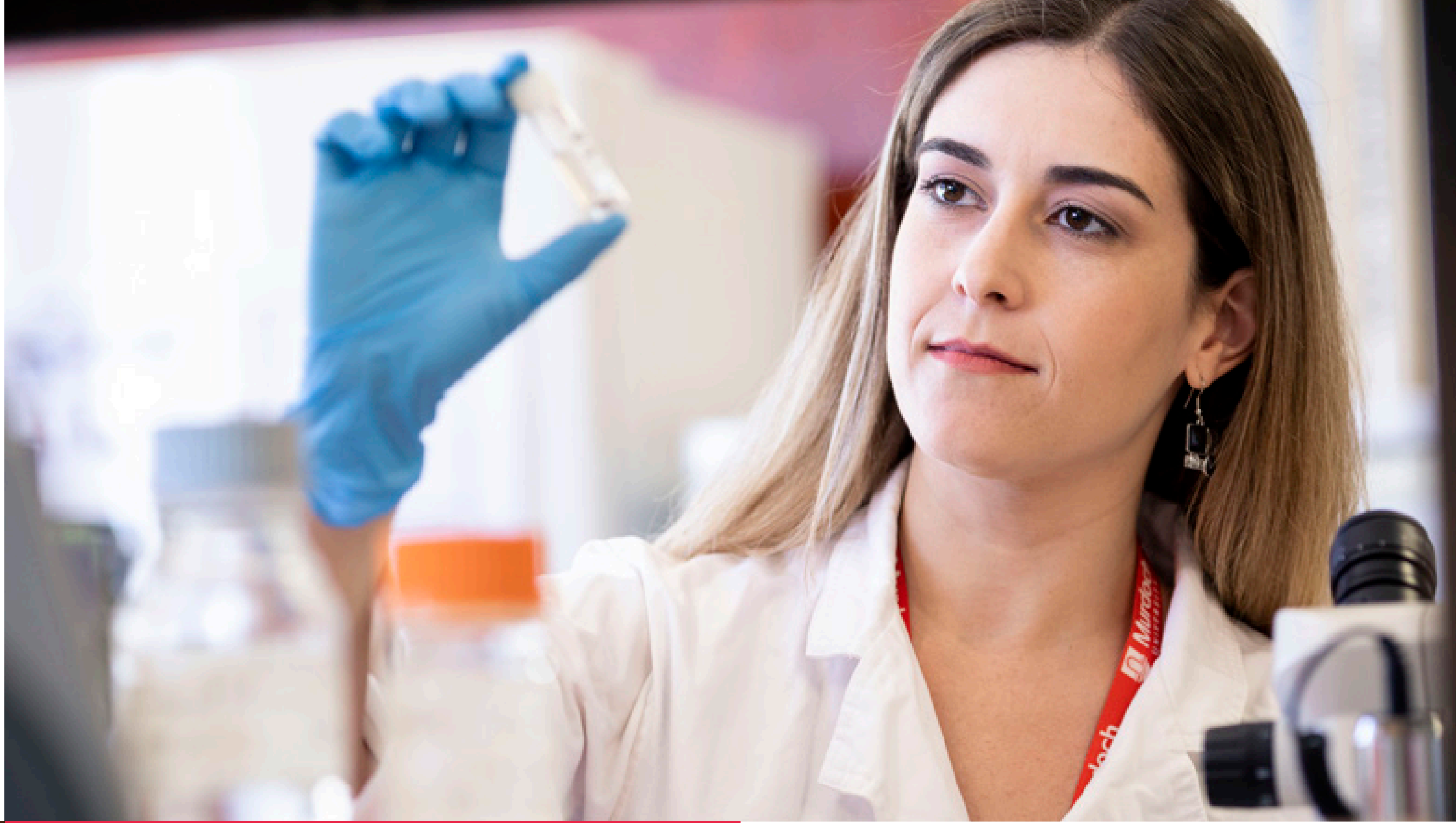

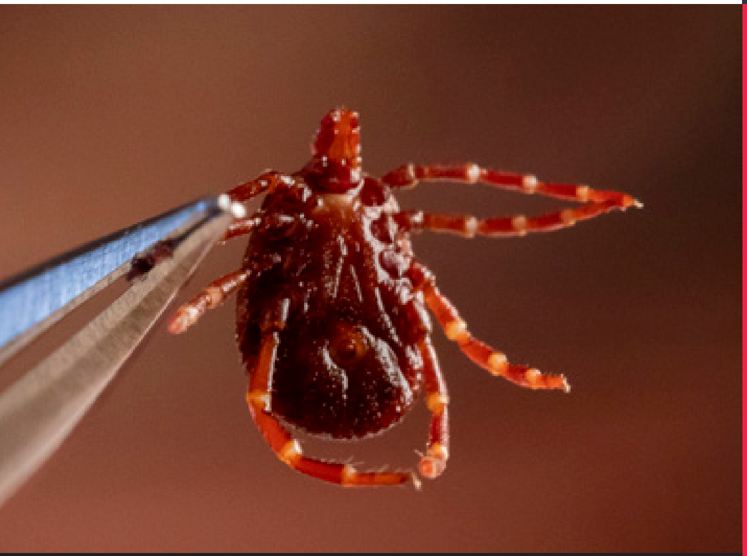

There will be no cost to you associated with participating in this research project and all information that we collect about you will be kept secure and confidential.

How can you help?

We are requesting that selected blood donors participate in this study as an important control group (i.e. healthy individuals, not bitten by a tick) for comparison with patients exposed to ticks. This means that if your age, sex and residency postcode match those of a tick-bitten patient who has enrolled in our study, you may be invited to participate.

If you are identified as matched control by one of our research team members in collaboration with the Australian Red Cross Lifeblood, we will greatly appreciate if you accept our invitation to enrol.

In medical research, control groups are crucial for discoveries to be achieved and validated.

Participation in the project is voluntary and involves:

- Enrolment and providing consent via our online study portal
- Completion of a brief questionnaire
- Providing a blood sample (which will be collected as part of the normal blood donation procedure)

Can anyone be part of this study?

If you are attending the Australian Red Cross Lifeblood to donate blood you may be invited to participate in this study.

You cannot join the project if:

1. You are pregnant.
2. You tend to bleed (i.e. if you are on blood-thinning medication or are a haemophiliac).
3. If you have a previous diagnosis of tick-related disease (e.g. rickettsiosis, spotted fever) or a chronic illness (such as Myalgic Encephalomyelitis, Chronic Fatigue Syndrome or a Lyme disease-like illness).
4. You are under 18 years old.

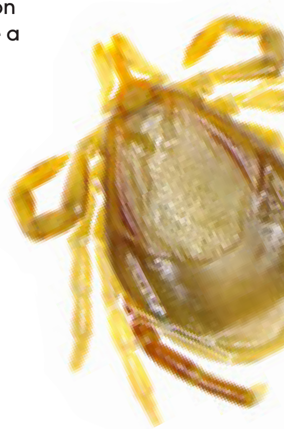

Photo credit: Siobhon Egan
