# Supplementary material for: The Troublesome Ticks Research Protocol: Developing a Comprehensive, Multidiscipline Research Plan for Investigating Human Tick-Associated Disease in Australia
Source: Pathogens. 2022 Nov 3;11(11):1290. doi: 10.3390/pathogens11111290 (PMC9694322; doi:10.3390/pathogens11111290)
Supplement: Supplementary file 1 [file pathogens-11-01290-s001.zip › Additional files/Figure S2.Participant recruitment flyer.pdf]

How do I avoid tick bites?

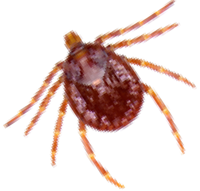

In gardens by:

- Avoiding overgrown or dense foliage and areas of deep leaf litter.
- Keeping lawns mown, removing long grass from garden borders.
- Regular trimming of bushes and shrubs and removal of low branches.
- Removing leaf and bark litter.
- Removing rubbish which could provide ticks with shelter.
- Ensuring these 'tick-reduced' areas are maintained in locations where children and pets are likely to access regularly.

Outdoors:

- Wearing suitable clothing that reduces the amount of bare skin that is exposed.
- Tuck long pants into socks and wear light coloured clothes so that any crawling ticks can be more easily seen and removed.
- Use commercially available insect/arthropod repellents.

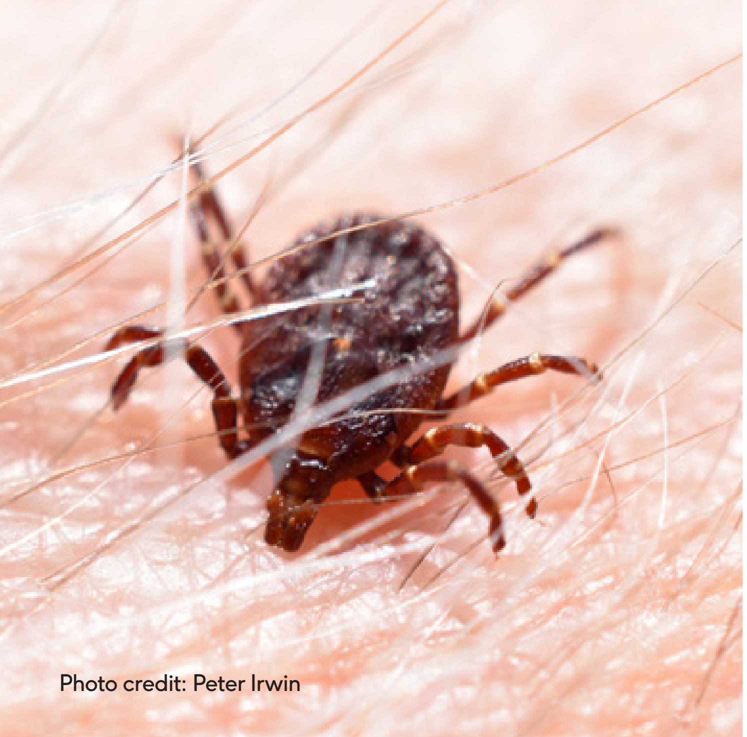

Photo credit: Peter Irwin

Murdoch UNIVERSITY

For more information, please contact our Project Manager on 1300 81 70 70, email DSCATT@rickettsialab.org.au or visit our website: tickstudy.murdoch.edu.au

This study has been approved by the Murdoch University Human Research Ethics Committee (permit 2019/124), the Northern Sydney Local Health District (permit 2019_ETH12032) and the ARC Lifeblood (permit 2019-20)

CRICOS Provider Code 00125J MCO0004789
Cover photo credit: Stephen Doggett

Research partners

NHMRC
Northern Beaches Hospital
Australian Rickettsial Reference Laboratory
MONASH University
THE UNIVERSITY OF WESTERN AUSTRALIA
THE UNIVERSITY OF QUEENSLAND AUSTRALIA
Australian Red Cross Lifeblood

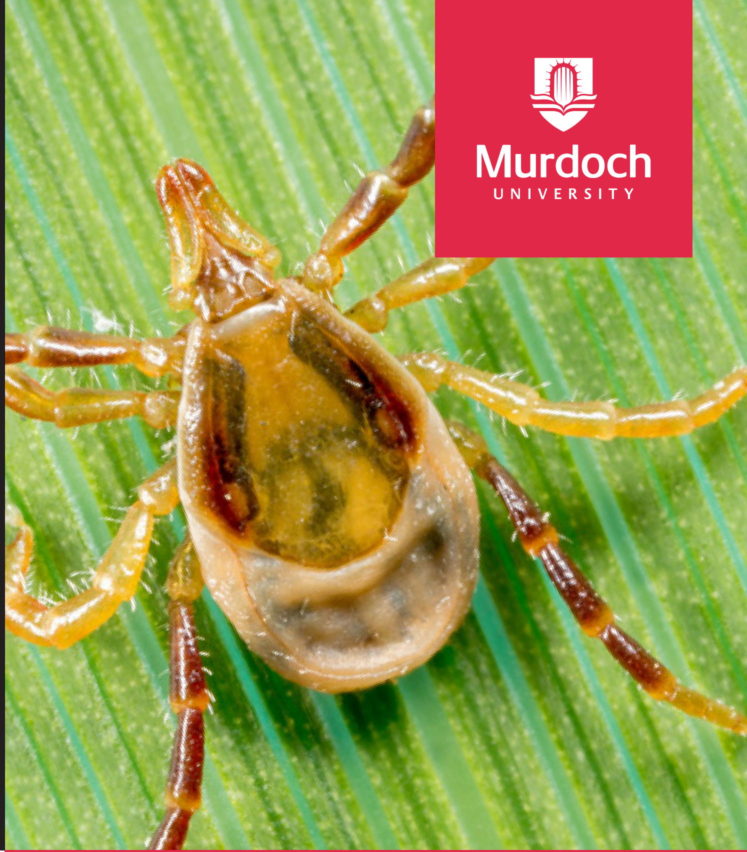

Murdoch UNIVERSITY

Have you been bitten by a tick?

Be part of ground-breaking medical research to help determine the cause(s) of Debilitating Symptom Complexes Attributed to Ticks (DSCATT) in Australia.

Phone 1300 81 70 70

Why This Study?

In Australia many people get bitten by ticks every year. There is uncertainty about why and how ticks make people unwell in Australia yet there are now thousands of patients suffering from fatigue, arthritis, chronic pain, headaches and psychological symptoms following a tick bite. These illnesses are known as “Debilitating Symptom Complexes Attributed to Ticks” (DSCATT). This research aims to find answers to questions about tick-associated illness in Australia.

Although this study is aimed at individuals who have just been bitten and currently have a tick attached to them, we would also like to hear from you if you’ve been bitten by a tick within the past 72 hours. Please do not discard the tick.

If this does not apply to you today, please keep this information in a safe place in case it’s ever needed in the future.

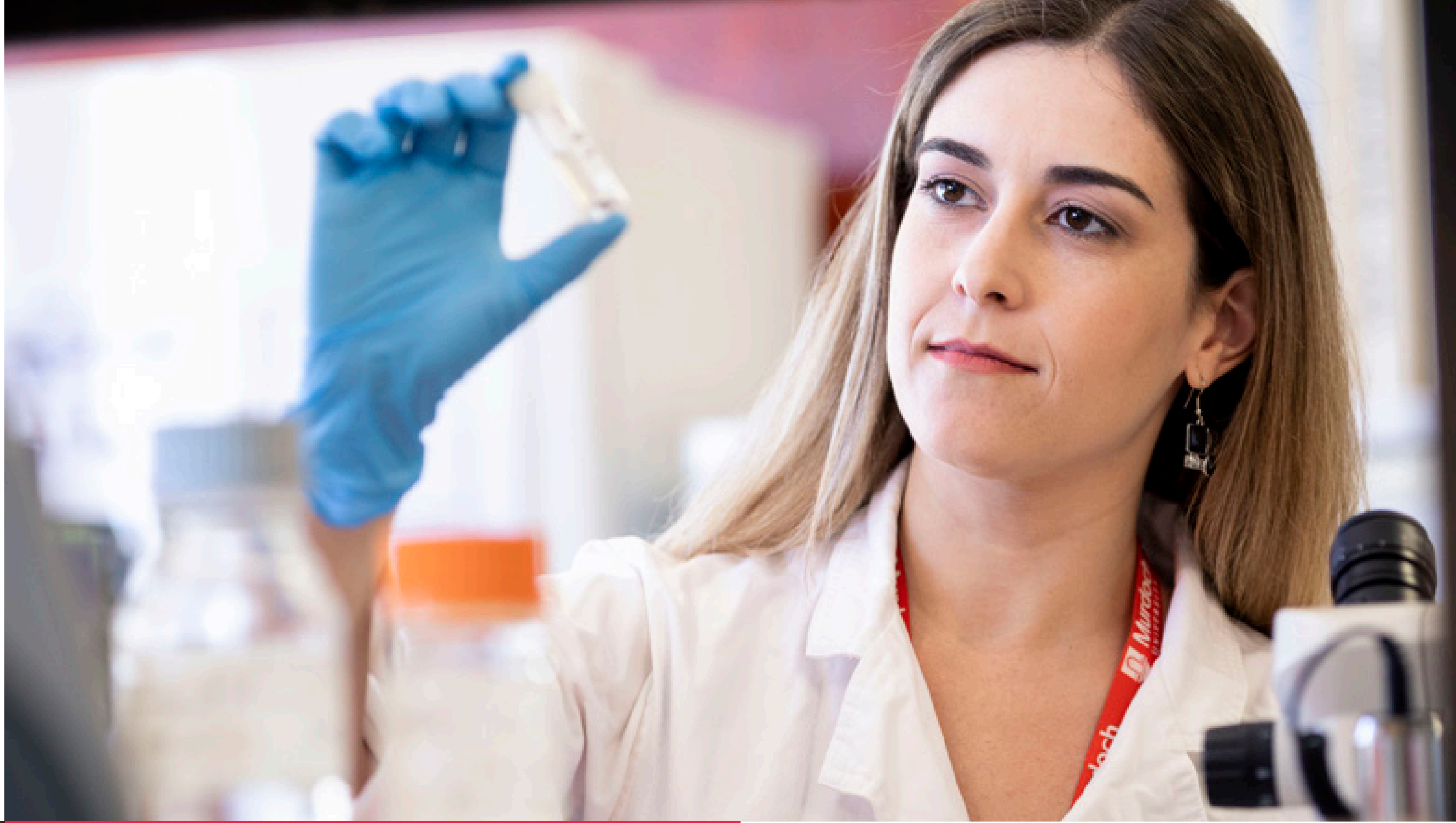

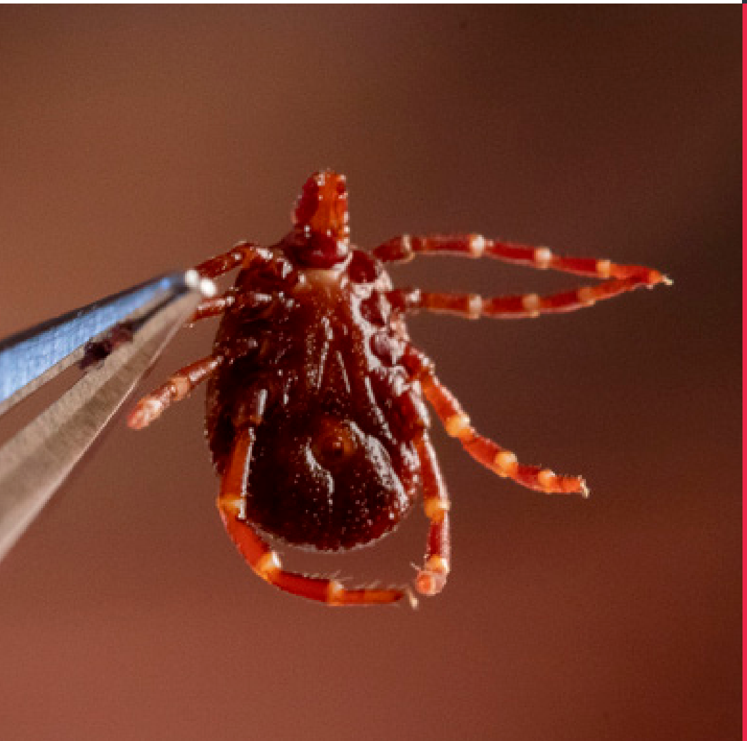

How can you help?

We urge you to participate in this study as it will provide important insights into the symptoms of DSCATT and help find ways to diagnose and treat the disease.

If you want to be part of this important research project, please ask the medical staff to enrol you when you’ve been bitten by a tick. If no medical staff are present, you can contact a Project Officer on 1300 81 70 70.

Participation in the project is voluntary and involves:

- Removal of the attached tick.
- Enrolment via our online study portal and completion of a brief questionnaire.
- A blood sample and simple skin biopsy taken when you see the doctor.
- Follow-up blood tests and health questionnaire.
- There will be no cost to you associated with participating in this research project and all information that we collect about you will be kept secure and confidential.

Please join the study if you have a tick attached to you now, have been bitten by a tick within the past 72 hours or if you get bitten by a tick in the future.

You cannot join the project if:

1. You are pregnant.
2. You tend to bleed (i.e. if you are on blood-thinning medication or are a haemophiliac).
3. If you have a previous diagnosis of tick-related disease (e.g. rickettsiosis, spotted fever) or a chronic illness (such as Myalgic Encephalomyelitis, Chronic Fatigue Syndrome or a Lyme disease-like illness).
4. You are under 18 years old.

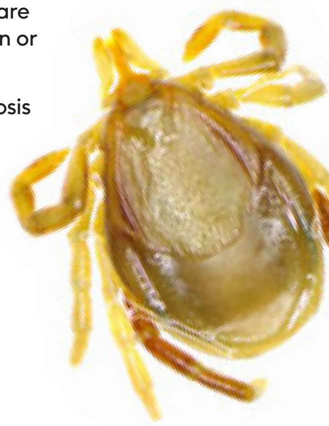

Photo credit: Siobhon Egan
