# Supplementary material for: The Troublesome Ticks Research Protocol: Developing a Comprehensive, Multidiscipline Research Plan for Investigating Human Tick-Associated Disease in Australia
Source: Pathogens. 2022 Nov 3;11(11):1290. doi: 10.3390/pathogens11111290 (PMC9694322; doi:10.3390/pathogens11111290)
Supplement: Supplementary file 1 [file pathogens-11-01290-s001.zip › Additional files/Figure S6.Biospecimen Processing Form.pdf]

Troublesome Ticks study

Biospecimen processing form

DATE OF ARRIVAL IN THE LAB (DD/MON/YYYY)		I__I I__I - I__I I__I – I__I I__I I__I I__I	
PARTICIPANT ID			
VISIT		I__I T0 I__I T1 I__I T2 I__I T3	
SPECIMENS RECEIVED<br>(Specify number of each biospecimen received)		I__I Tick I__IBiopsy I__IPAXgene<br>I__I Heparin I__IEDTA I__I SST	
Tube lot number		Lithium heparin: ____________________________<br>PAXgene: ____________________________	
Coloration of sample:<br>Y = Yellow; C = Cloudy; P = Pink; R = Red		Plasma-Hep I__I Y I__I C I__I P I__I R<br>Plasma-EDTA I__I Y I__I C I__I P I__I R<br>Serum I__I Y I__I C I__I P I__I R	
Time of spin		Plasma-Hep I__I I__I : I__I I__I hrs<br>Plasma-EDTA (1st) I__I I__I : I__I I__I hrs<br>Plasma-EDTA (2nd) I__I I__I : I__I I__I hrs<br>Serum I__I I__I : I__I I__I hrs	
Volume of PLAS1	Volume of PLAS2	Volume of PLAS3	Volume of PLAS (EDTA)
I__I I__I I__I ul	I__I I__I I__I ul	I__I I__I I__I ul	I__I I__I I__I ul
Time of freeze		PLAS 1-4 I__I I__I : I__I I__I hrs<br>PAXgene I__I I__I : I__I I__I hrs	
RESEARCH STAFF INITIALS	Sample receival at<br>I__I MU I__I TKI	I__I I__I I__I	
	Sample processing at<br>I__I MU I__I TKI	I__I I__I I__I	
COMMENTS OR DEVIATIONS TO PROTOCOL			

PLEASE RECORD DATA ON REDCap, then ARCHIVE THIS FORM
