# Supplementary material for: The Troublesome Ticks Research Protocol: Developing a Comprehensive, Multidiscipline Research Plan for Investigating Human Tick-Associated Disease in Australia
Source: Pathogens. 2022 Nov 3;11(11):1290. doi: 10.3390/pathogens11111290 (PMC9694322; doi:10.3390/pathogens11111290)
Supplement: Supplementary file 1 [file pathogens-11-01290-s001.zip › Additional files/Figure S1.Participant recruitment poster.pdf]

Participants Required

for Research Study on Tick Bites in Australia.

Murdoch UNIVERSITY

If you have a tick attached to you now, have been bitten by a tick within the past 72 hours or get a tick bite in the future, you may be able to assist our researchers on the Troublesome Ticks Project.

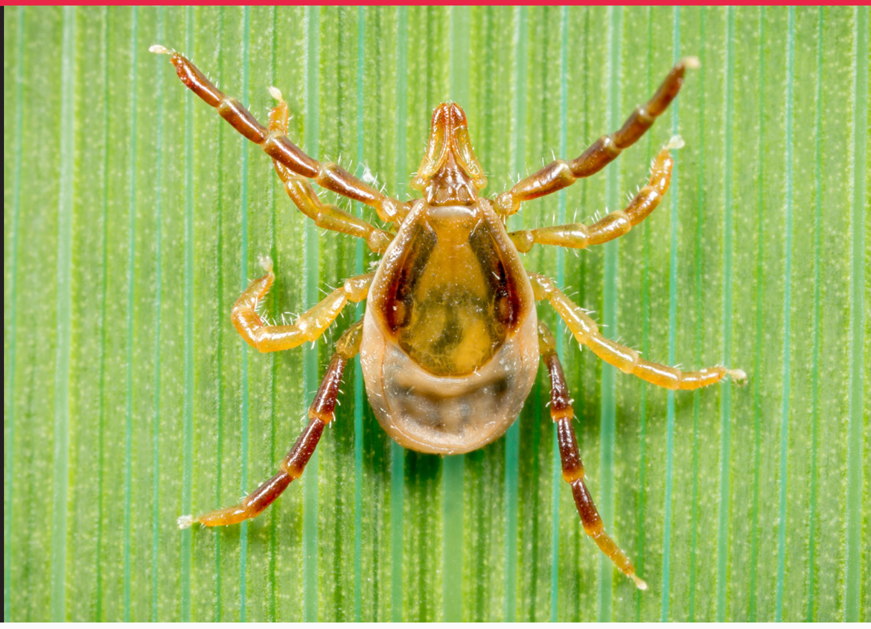

How you can help

Although we know that diseases can be transmitted through Australian tick bites, there is much debate as to what is causing the debilitating symptom complexes attributed to ticks (DSCATT) seen in some patients. Your participation in our study will provide valuable information which may assist us in detecting the causes of DSCATT. Any reasonable out-of-pocket expenses will be reimbursed.

Participation includes:

- Removal of the attached tick
- Enrolment via our study portal and completion of some questions
- A blood sample and simple skin biopsy
- Follow-up blood tests and health questionnaire.

Interested?

If you are interested in participating, please ask the medical staff at your local clinic or hospital to enrol you in our study now.

For more information, please contact our Project Officer on 1300 817070, email DSCATT@rickettsialab.org.au or head to the website: tickstudy.murdoch.edu.au

Murdoch University | The University of Queensland Australia | Monash University | Northern Beaches Hospital | NHMRC | Australian Red Cross Lifeblood | The University of Western Australia | Australian Rickettsial Reference Laboratory

This study has been approved by the Murdoch University Human Research Ethics Committee (permit 2019/124), the Northern Sydney Local Health District (permit 2019_ETH12032) and the ARC Lifeblood (permit 2019-20). CRICOS provider code: 00125J | MCO0004789 | Photo credit: Stephen Doggett

1300 817070 tickstudy.murdoch.edu.au
