# Supplementary material for: The Troublesome Ticks Research Protocol: Developing a Comprehensive, Multidiscipline Research Plan for Investigating Human Tick-Associated Disease in Australia
Source: Pathogens. 2022 Nov 3;11(11):1290. doi: 10.3390/pathogens11111290 (PMC9694322; doi:10.3390/pathogens11111290)

GP1- TICK-BITTEN PATIENTS (Tick Collection and Sampling Kit)

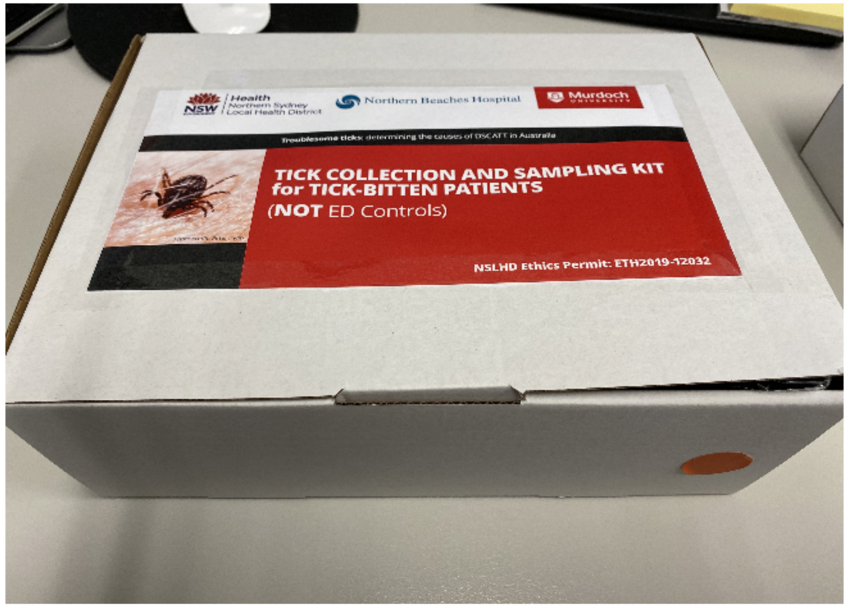

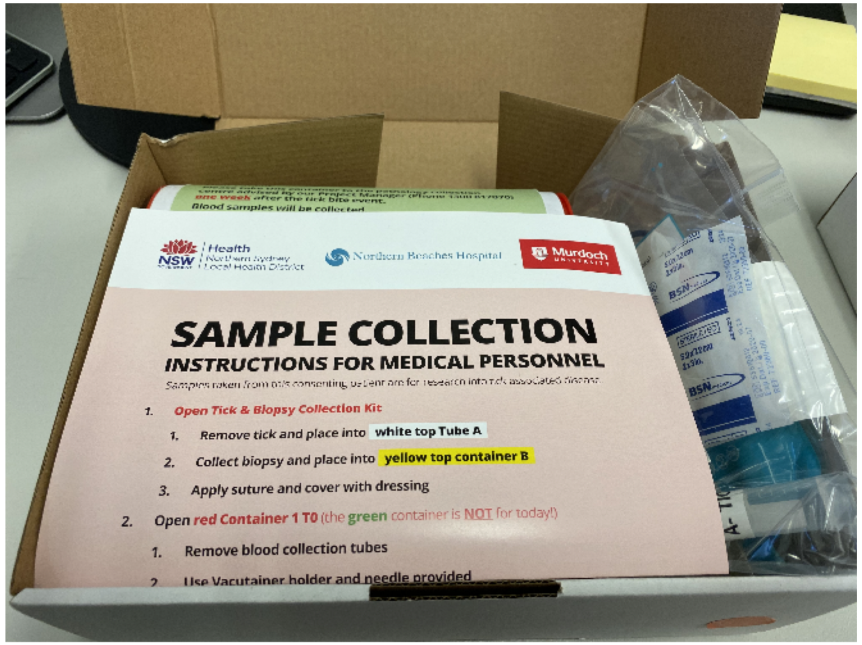

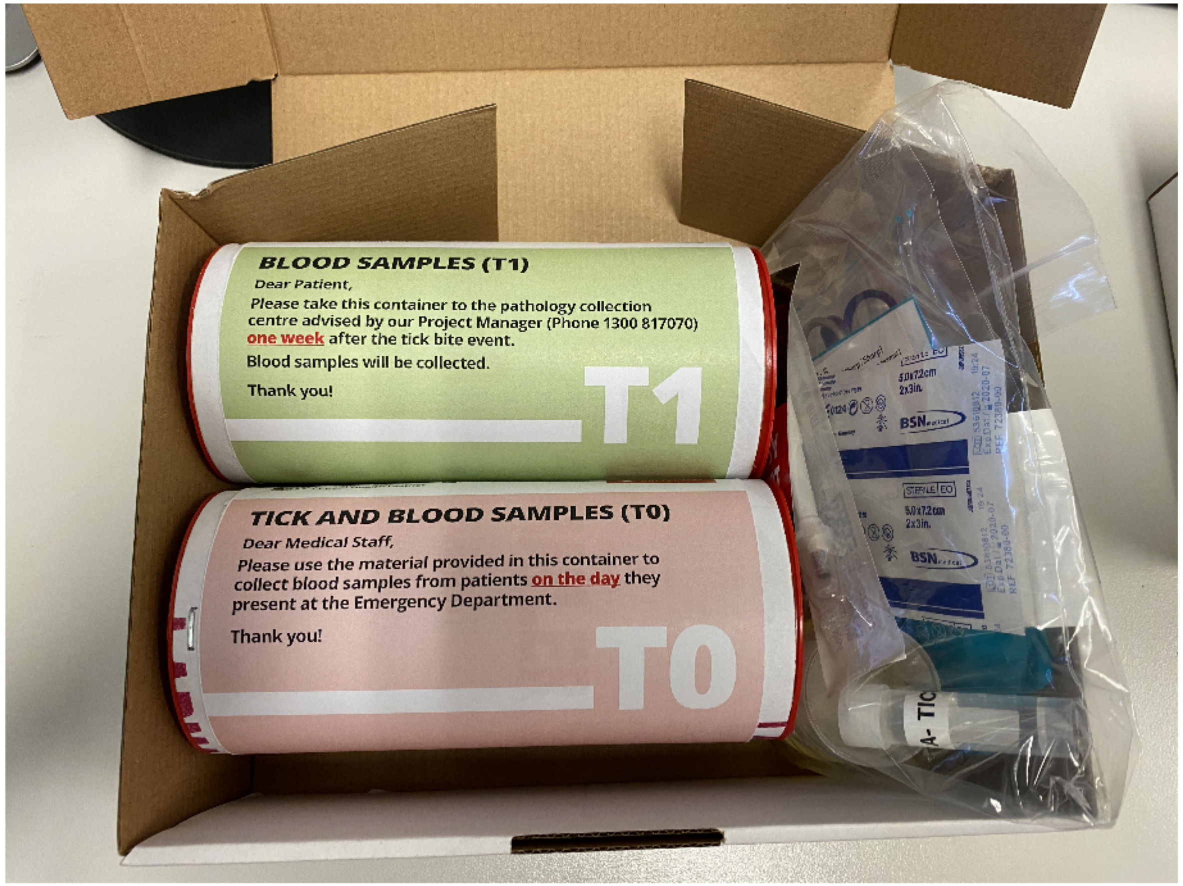

GP1- TICK-BITTEN PATIENTS (Tick and Biopsy Collection Kit)

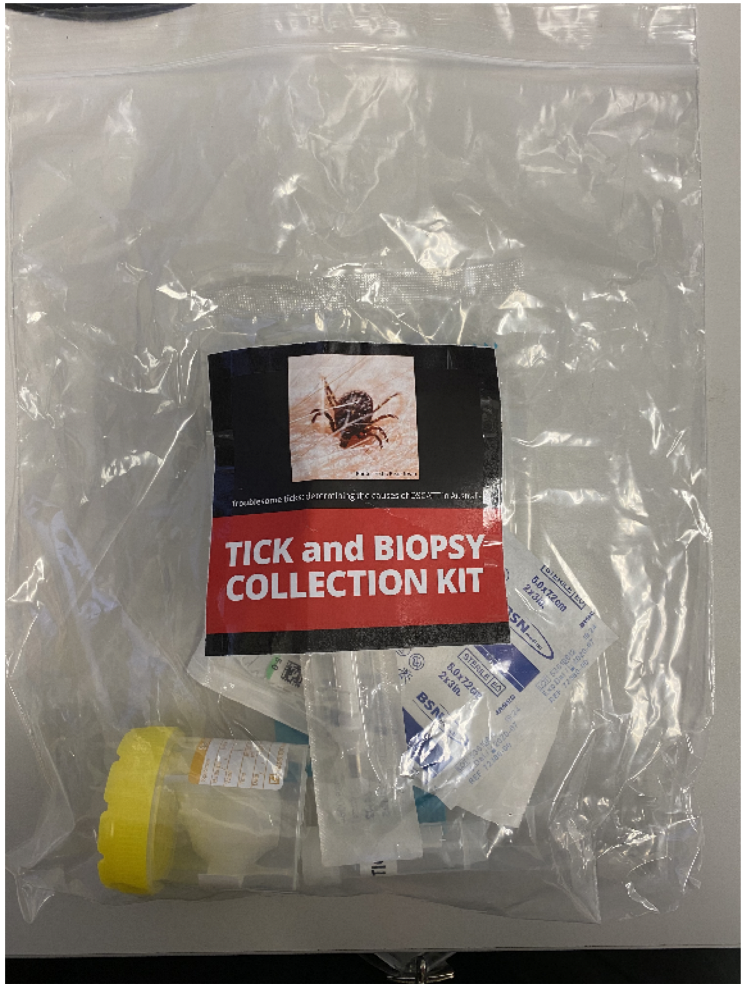

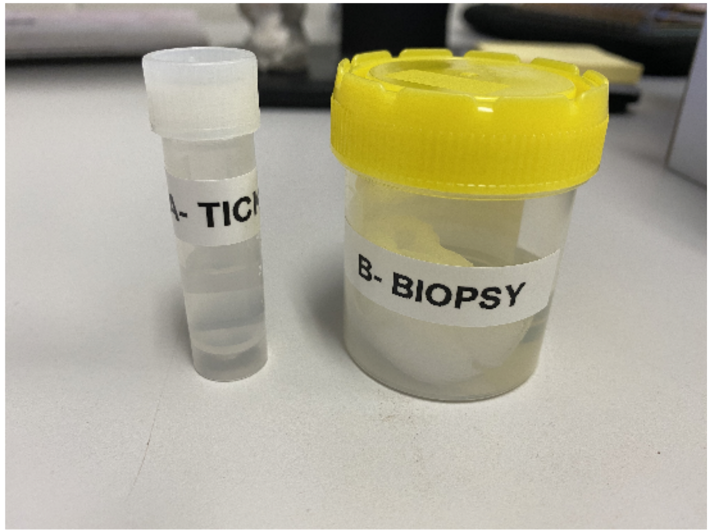

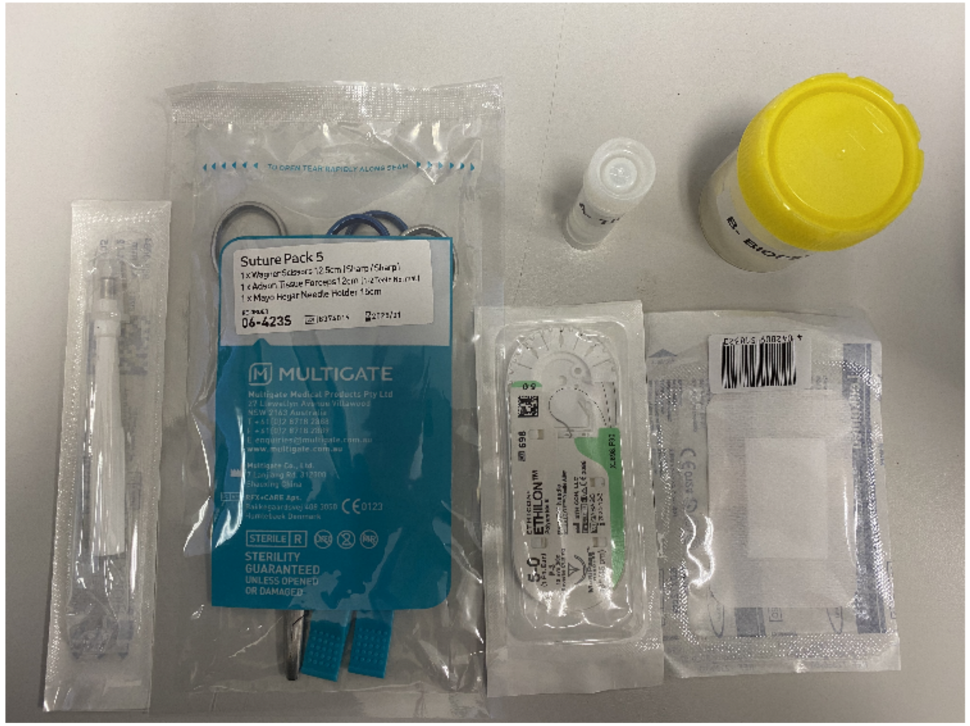

GP1- TICK-BITTEN PATIENTS (Blood Sampling Kit – T_0)

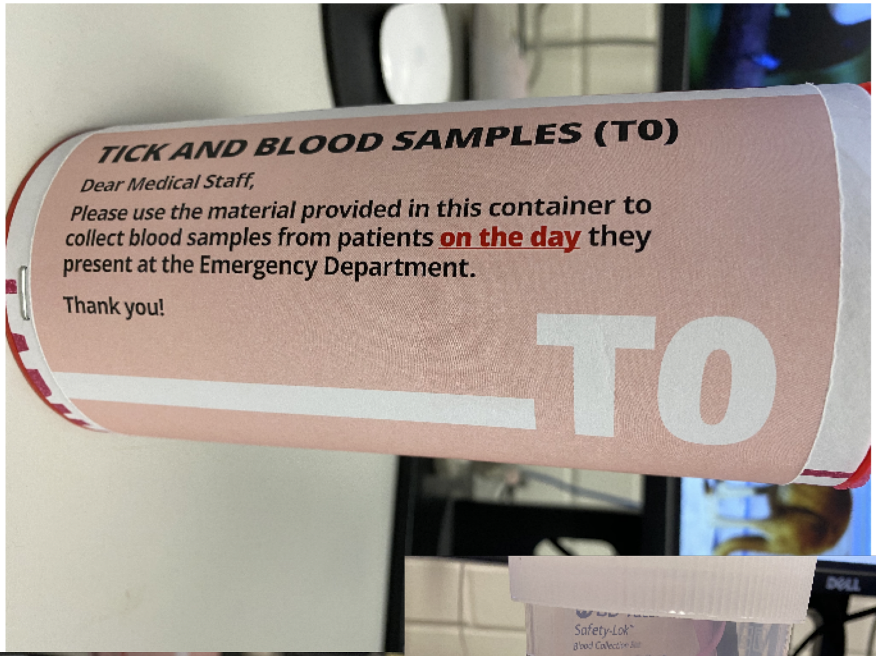

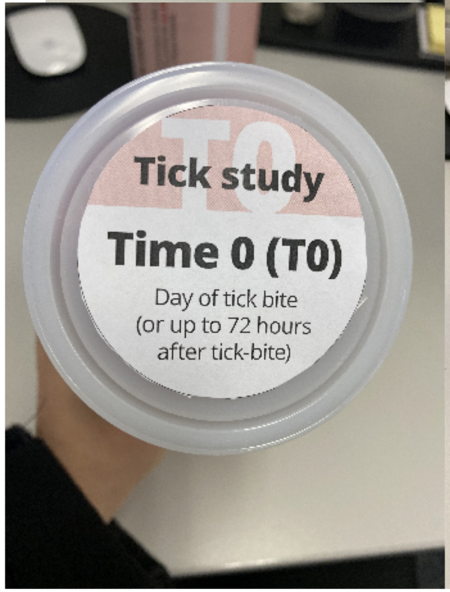

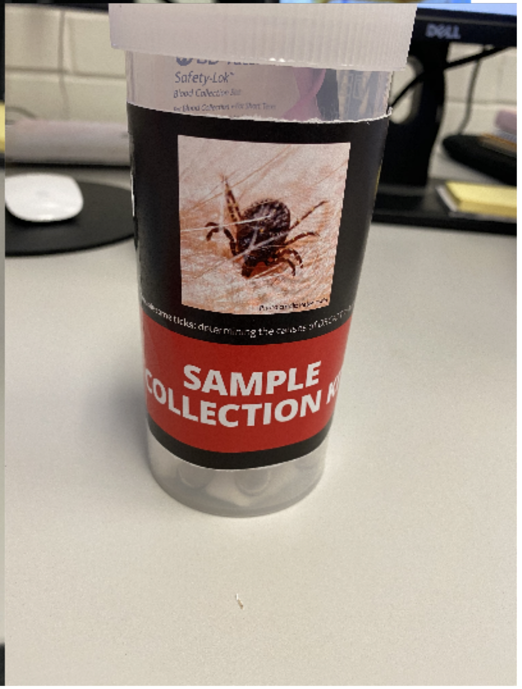

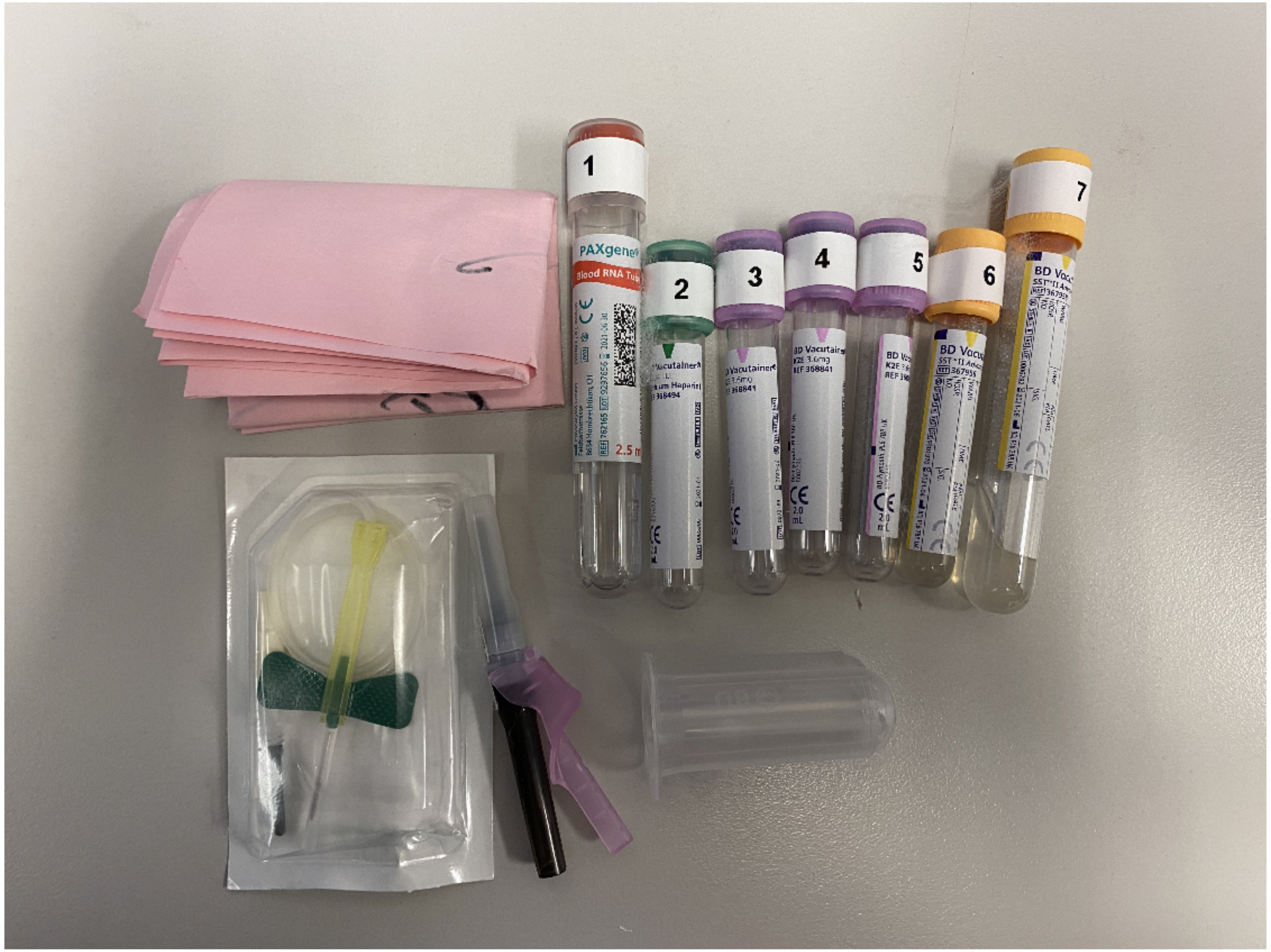

GP1- TICK-BITTEN PATIENTS (Blood Sampling Kit – T_1)

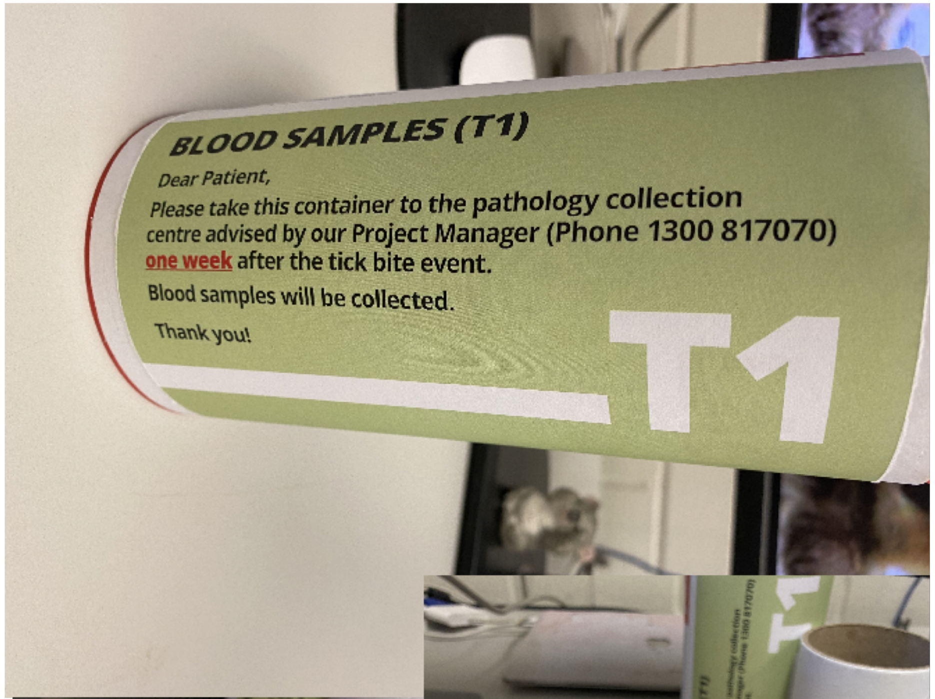

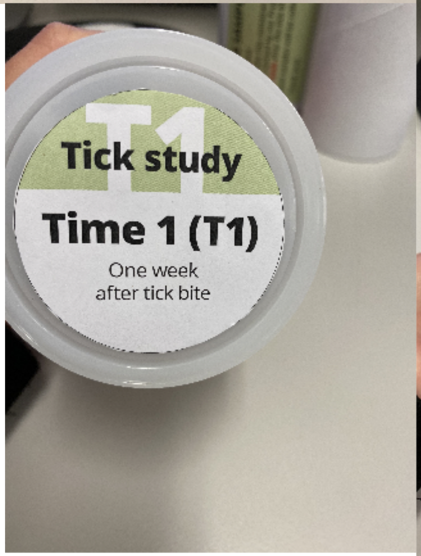

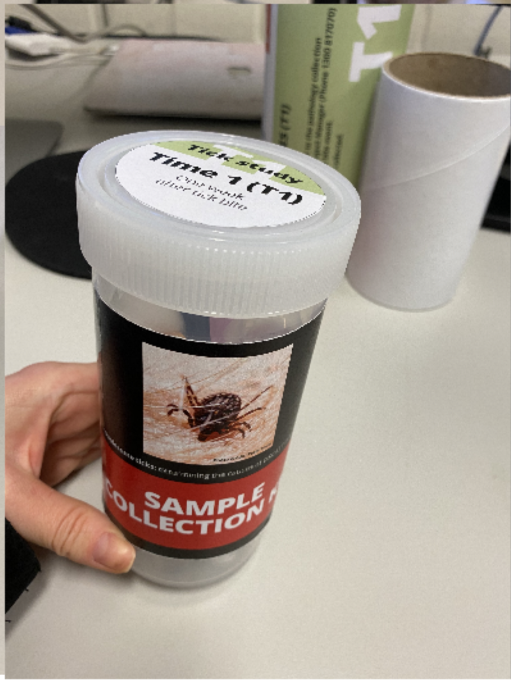

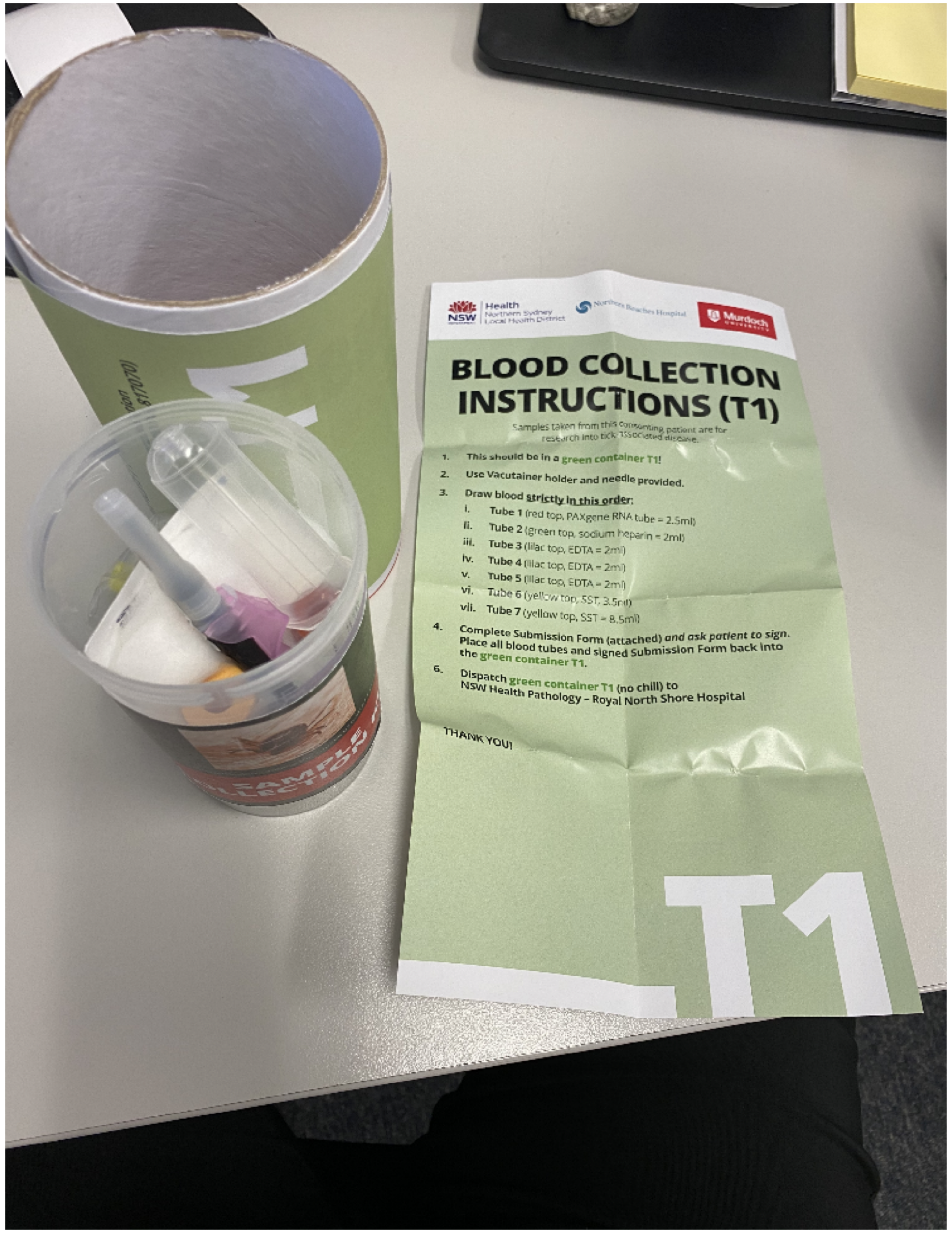

GP2- ED CONTROLS (Blood Sampling Kit)

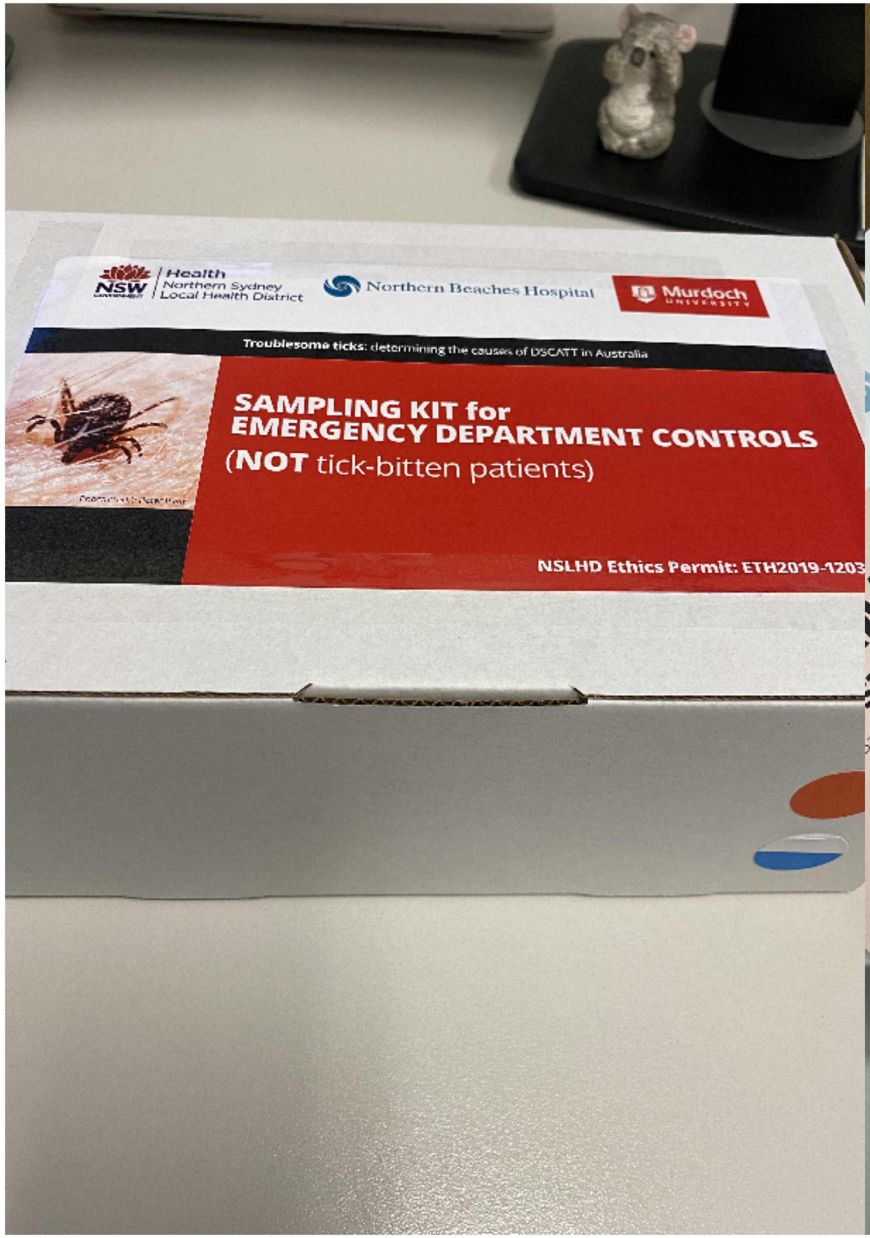

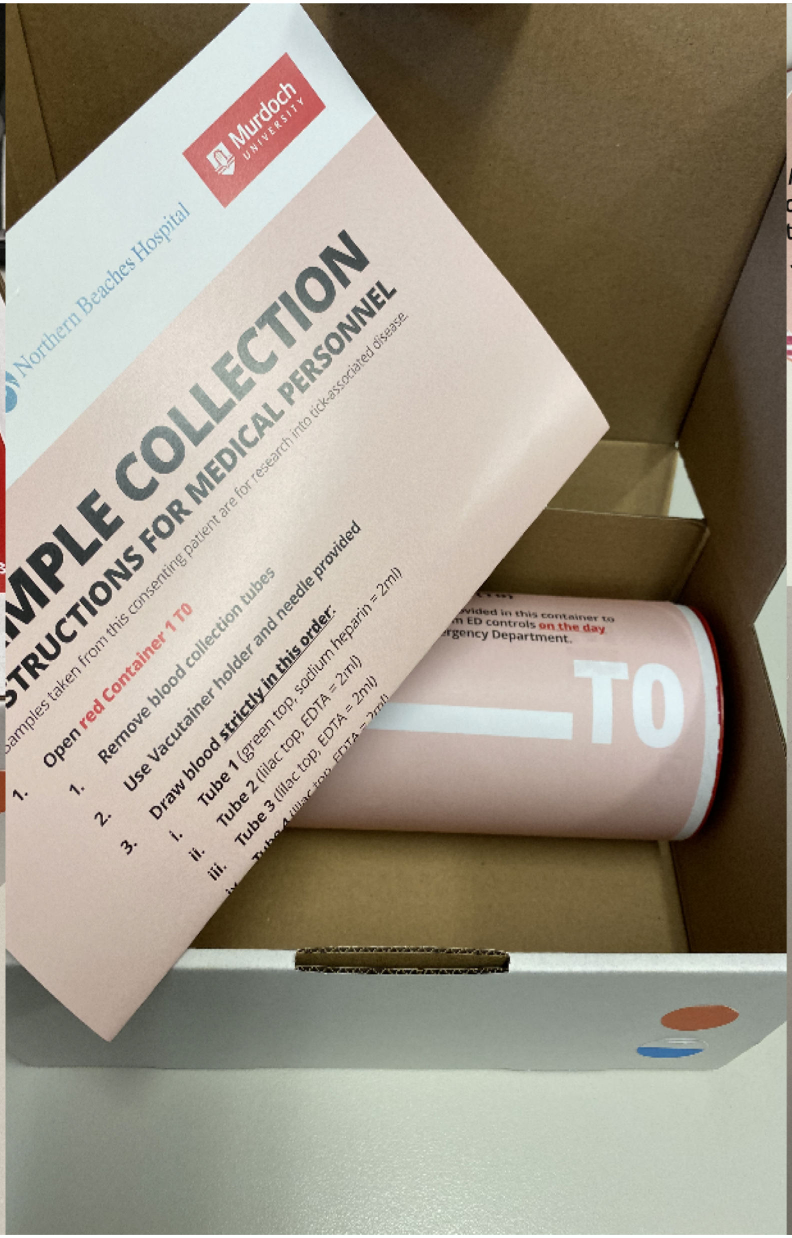

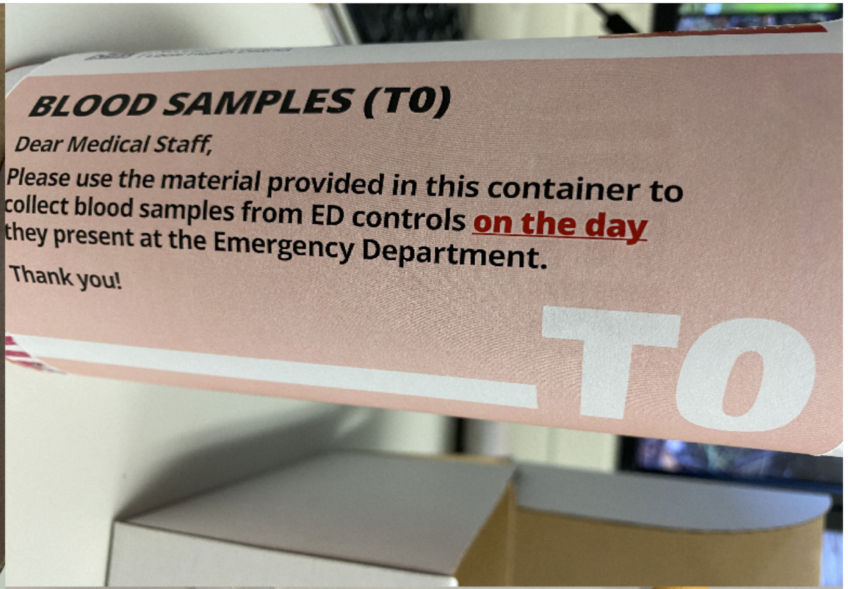

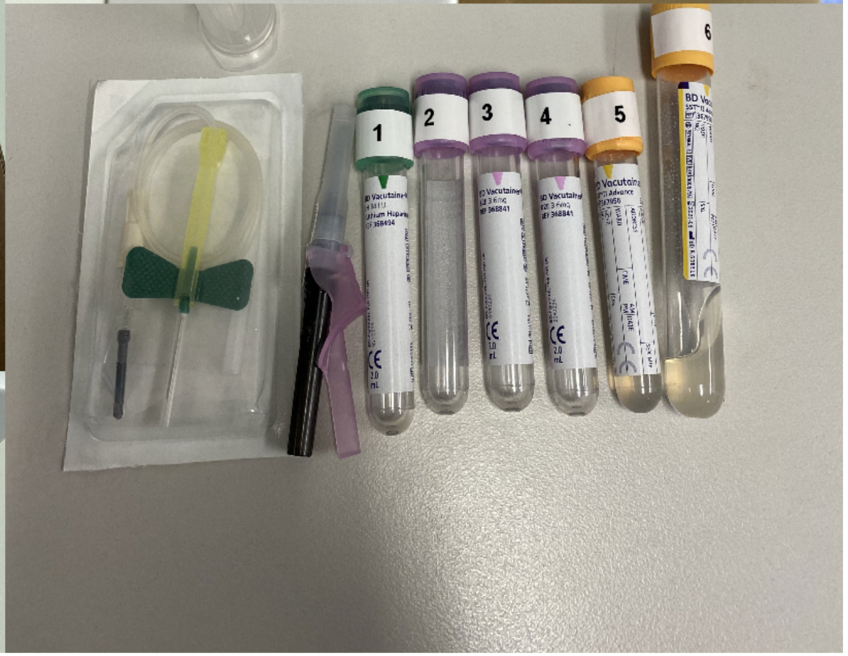

Supplement: Supplementary file 1 [file pathogens-11-01290-s001.zip › Additional files/Figure S5.Sampling kits design.pdf]
