# Supplementary material for: The Troublesome Ticks Research Protocol: Developing a Comprehensive, Multidiscipline Research Plan for Investigating Human Tick-Associated Disease in Australia
Source: Pathogens. 2022 Nov 3;11(11):1290. doi: 10.3390/pathogens11111290 (PMC9694322; doi:10.3390/pathogens11111290)
Supplement: Supplementary file 1 [file pathogens-11-01290-s001.zip › Additional files/Figure S4.Troublesome Ticks Portal.pdf]

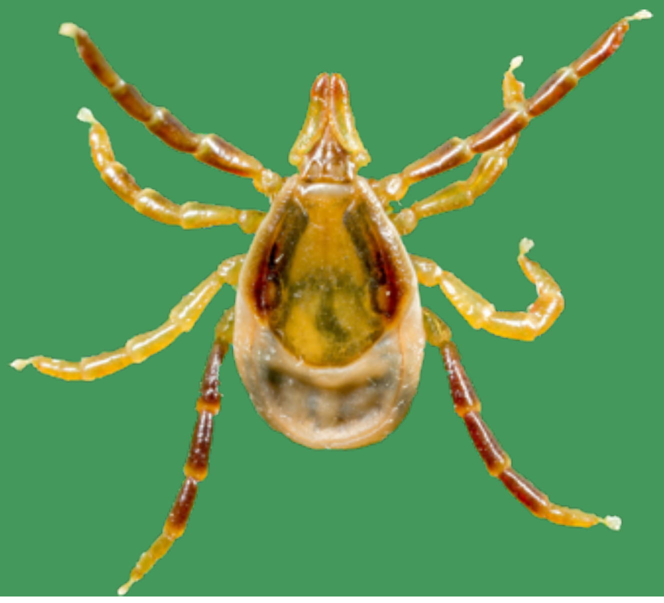

PARTICIPANT PORTAL

Welcome!

Thanks for volunteering. Your participation in this study will help us identify the illnesses that affect thousands of Australians every year. It could be YOUR data that makes all the difference!

NB: Please don't press the 'back' button while using this Participant Portal

SIGN UP / SIGN IN

Murdoch University

CONTACT US

Troublesome Ticks Research

1300 817070

DSCATT@rickettsialab.org.au

TEQSA number: PRV12163 CRICOS Code: 00125J Copyright & Disclaimer Privacy © Murdoch University

Made by The App Workshop
